# Routine OGTT: A Robust Model Including Incretin Effect for Precise Identification of Insulin Sensitivity and Secretion in a Single Individual

**DOI:** 10.1371/journal.pone.0070875

**Published:** 2013-08-29

**Authors:** Andrea De Gaetano, Simona Panunzi, Alice Matone, Adeline Samson, Jana Vrbikova, Bela Bendlova, Giovanni Pacini

**Affiliations:** 1 Institute of System Analysis and Informatics (IASI) “A. Ruberti”, National Research Council (CNR), Rome, Italy; 2 Laboratoire MAP5, Universite’ Paris Descartes, Paris, France; 3 Department of Molecular Endocrinology, Institute of Endocrinology, Prague, Czech Republic; 4 Metabolic Unit, Institute of Biomedical Engineering (ISIB), National Research Council (CNR), Padua, Italy; Manchester University, United Kingdom

## Abstract

In order to provide a method for precise identification of insulin sensitivity from clinical Oral Glucose Tolerance Test (OGTT) observations, a relatively simple mathematical model (Simple Interdependent glucose/insulin MOdel SIMO) for the OGTT, which coherently incorporates commonly accepted physiological assumptions (incretin effect and saturating glucose-driven insulin secretion) has been developed. OGTT data from 78 patients in five different glucose tolerance groups were analyzed: normal glucose tolerance (NGT), impaired glucose tolerance (IGT), impaired fasting glucose (IFG), IFG+IGT, and Type 2 Diabetes Mellitus (T2DM). A comparison with the 2011 Salinari (COntinuos GI tract MOdel, COMO) and the 2002 Dalla Man (Dalla Man MOdel, DMMO) models was made with particular attention to insulin sensitivity indices IS_COMO_, IS_DMMO_ and *k_xgi_* (the insulin sensitivity index for SIMO). ANOVA on *k_xgi_* values across groups resulted significant overall (P<0.001), and post-hoc comparisons highlighted the presence of three different groups: NGT (8.62×10^−5^±9.36×10^−5^ min^−1^pM^−1^), IFG (5.30×10^−5^±5.18×10^−5^) and combined IGT, IFG+IGT and T2DM (2.09×10^−5^±1.95×10^−5^, 2.38×10^−5^±2.28×10^−5^ and 2.38×10^−5^±2.09×10^−5^ respectively). No significance was obtained when comparing IS_COMO_ or IS_DMMO_ across groups. Moreover, *k_xgi_* presented the lowest sample average coefficient of variation over the five groups (25.43%), with average CVs for IS_COMO_ and IS_DMMO_ of 70.32% and 57.75% respectively; *k_xgi_* also presented the strongest correlations with all considered empirical measures of insulin sensitivity. While COMO and DMMO appear over-parameterized for fitting single-subject clinical OGTT data, SIMO provides a robust, precise, physiologically plausible estimate of insulin sensitivity, with which habitual empirical insulin sensitivity indices correlate well. The *k_xgi_* index, reflecting insulin secretion dependency on glycemia, also significantly differentiates clinically diverse subject groups. The SIMO model may therefore be of value for the quantification of glucose homeostasis from clinical OGTT data.

## Introduction

Metabolic conditions related to glucose tolerance disorders exist in several distinct forms, such as Type 2 Diabetes Mellitus (T2DM), Impaired Glucose Tolerance (IGT) and Impaired Fasting Glucose (IFG). In order to prevent and treat such disorders, early diagnosis of glucose intolerance is of crucial importance, since a deterioration of beta-cell function can determine the conversion of impaired glucose metabolism (IGM) to diabetes [1]. However, it has been shown that also subjects with normal glucose metabolism can show elusive impairment of beta-cell function [2]. Therefore, identification and characterization of altered beta-cell function can help understand and potentially prevent disease development [3] [4].

The euglycemic-hyperinsulinemic clamp technique is widely considered to be the reference method for the assessment of insulin sensitivity. This procedure, however, is complicated, experimentally demanding, and costly: its use outside of specialized research centers is impractical. Moreover, clinical research involving the assessment of metabolic parameters has moved from small patient samples to large trials, thus making the use of the clamp technique even more unrealistic. Alternative methods applicable to large studies have been proposed. Among these, the Intravenous Glucose Tolerance Test (IVGTT) is experimentally easier, but the need of frequent blood sampling makes its application to a large number of patients difficult. Oral tests, such as the Mixed Meal and the Oral Glucose Tolerance Test (MMTT, OGTT), in addition to being simpler, are also more reliable because the oral administration triggers a physiological secretion of glucose regulating hormones, such as gastrointestinal incretins [5]. The MMTT and OGTT are in fact more physiological tests, mimicking habitual carbohydrate intake.

The OGTT is a very common test in medical practice: it consists of administering glucose orally and detecting, by means of a few blood samples, how rapidly it is absorbed into and then cleared from the blood stream. For its simplicity, it is a method suitable for large studies assessing insulin secretion and action. As regards insulin sensitivity, several attempts have been made to obtain surrogate measurements [6]–[10]; however, it would seem logical to use a suitable “patient-tailored” mathematical model to extract from observed concentrations as much information as possible on insulin secretion and sensitivity.

An ideal model ought to be simple and devoid of too many arbitrary assumptions. Arbitrary mathematical constructs have been used in the past to overcome the inherent lack of robustness of some models, encountered when trying to estimate too many parameters from relatively small data sets. Model assumptions should however be physiologically plausible and not simply represent *ad hoc* numerical simplification shortcuts. A good usable model should therefore be simple, with as few free parameters as practicable and should remain pertinent by directly incorporating the variables of physiological and clinical interest [8].

The aim of the present study is the development of a relatively simple mathematical model for the OGTT, which nonetheless coherently incorporates commonly accepted physiological assumptions, providing a patient-oriented approach for the identification of insulin sensitivity and secretion.

The present model extends and integrates several previous contributions [10]–[13], with the stated goal of achieving a compact, clinically applicable formulation, with good physiological plausibility and without sacrifice in adaptation to observations. In Dalla Man et al. [10], the classic minimal model of glucose kinetics [14] was coupled with a parametric model of the rate of glucose appearance (Ra), described in piecewise linear fashion. Although data fitting was generally good when that model of Ra was applied to our data, we found that the model was neither numerically robust (with large variability in parameter estimates), nor physiologically convincing: a piecewise-linear approximation of glucose gastrointestinal absorption needs many parameters and, above all, uses noisy observations as theoretical, error-free pivotal points, which is statistically inconsistent. Salinari et al. [13] proposed a new version of the OGTT minimal model [15], [16], where gastric emptying, the transport of glucose along the intestinal tract and its absorption from gut lumen into portal blood predicted the time course of glucose Ra in terms of parameters with a direct physiological meaning. The Salinari model also provided an expression for the release of incretin hormones as related to glucose transit into gut lumen. While this formulation has physiological value, its identification requires fitting the model to OGTT glucose and GLP-1 data, which are not collected during standard clinical practice. Moreover, while the Salinari model does address the physiological plausibility of the prediction of glucose Ra, the model lacks a representation of insulin dynamics, using instead interpolated noisy observations as a forcing function for plasma glucose dynamics. Interpolating noisy observations in order to represent the expected values of a state variable is incorrect and opens the door to potentially very misleading parameter estimation results: see elsewhere [17], [18] a complete discussion of this issue in the present glucose-modeling context.

For the purpose of the present work, computations for the Dalla Man and Salinari models were performed in Matlab and in R, both interfacing with C routines. The Salinari model was implemented in the Matlab environment and the “interp1” function was used (piecewise linear interpolation). Predictions for the Dalla Man Model were obtained in C++ interfacing with the R environment, and as before, a linear interpolation was used.

We present here a new model (Simple Interdependent glucose/insulin MOdel SIMO) where, on one hand, insulin secretion from beta-cells after glucose intake is represented by a mathematical formulation of the theoretical predicted insulin concentrations rather than by the noisy observations themselves, and where, on the other hand, the glucose rate of appearance is derived by absorption of glucose along the gastrointestinal tract, represented by a sequence of three compartments, as a simplification of the continuous one-dimensional process presented in Salinari et. al. [13].

The model can be rapidly implemented in standard computational software packages (*e.g.* Matlab, R, Scilab, Octave, *etc*.) and its computation is freely available via internet at the page http://biomat1.iasi.cnr.it/gemini/ogtt/. In the present work we have applied this model to data from 78 patients from five different glucose tolerance groups: Normal Glucose Tolerance (NGT), IGT, IFG, IFG+IGT and T2DM, showing that model parameters, identified on each subject, reflect accurately and informatively the underlying physiological status in the different conditions examined. We also compared the new model’s performance with standard empirical and model-based indices of insulin sensitivity.

## Materials and Methods

### Model Development

During model development, we studied the use of a series of glucose absorption compartments, that is, a number of sections through which glucose sequentially transits, before becoming available in plasma: no clear advantage was obtained if more than three compartments, in addition to the Stomach, were considered.

Therefore, four compartments, corresponding indicatively to Stomach, Jejunum and Ileum, plus a delay compartment between Jejunum and Ileum, were included in the model. Glucose entry into the gut causes the release of incretin hormones, which have an effect on insulin release. Incorporating the incretin mechanism proved to be essential for model performance. The incretin effect was assumed to stem from glucose content in the Jejunum and Ileum. We also considered modeling stomach emptying by means of a nonlinear dynamics (results not shown), but results were no better than with the linear version. Incorporating in the model both an incretin mechanism and a limited power progression of pancreatic insulin release with increasing glycemias turned out to be fundamental in fitting data: a good performance of the model strongly depended on both these features.

### Model Structure

**Figure 1:**
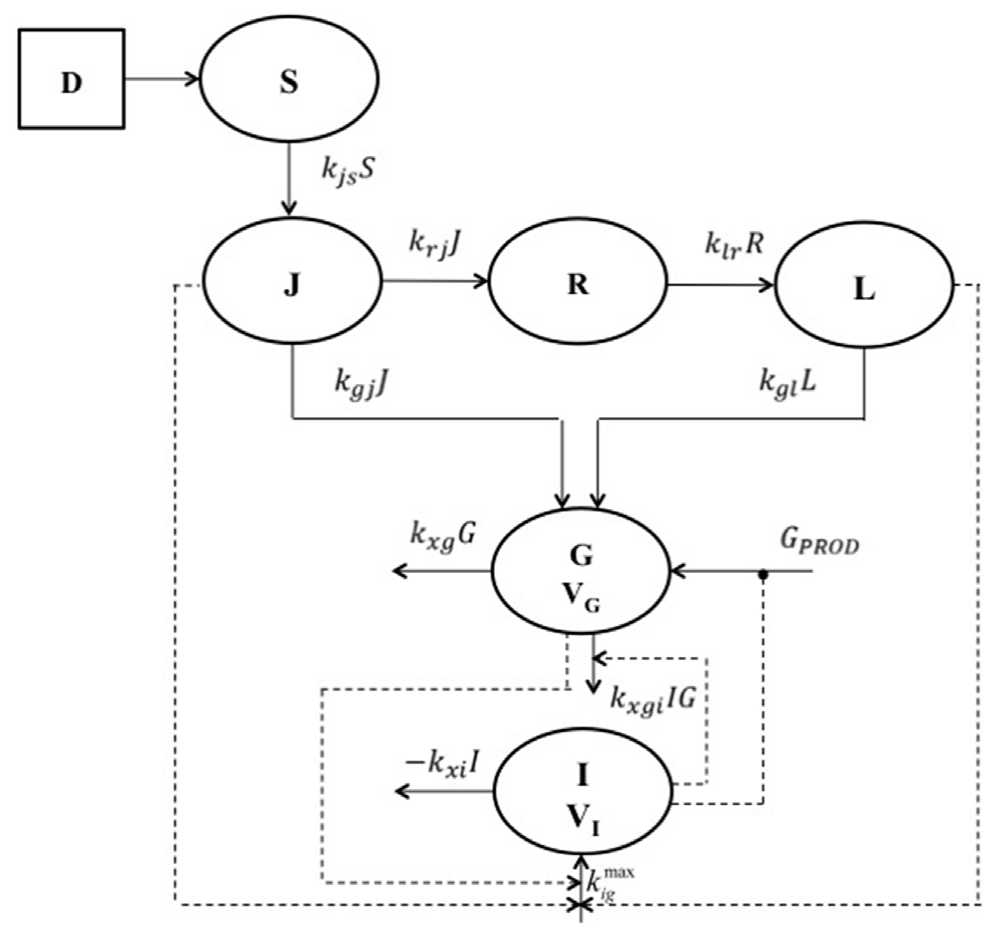
Block diagram of the model. Schematic representation of the six-compartment model. D is the orally administered quantity of glucose. S represents the quantity of glucose in the stomach while J, R and L represent the glucose content in the jejunum, in a delay compartment and in the Ileum respectively. G indicates the compartment for the plasma glucose concentration and I indicates the insulin plasma concentration. Measurements were taken for plasma glucose and insulin concentrations. Continuous lines represent entry or exit fluxes while dotted lines represent stimulation (arrows) or inhibition (black circles) mechanisms.



A block diagram of the model is shown in Figure 1, schematically illustrating compartments and their interactions. The model consists of the following six compartmental ordinary differential equations:

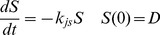
(1)

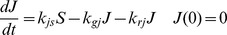
(2)
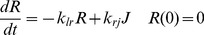
(3)

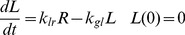
(4)

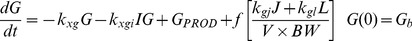
(5)

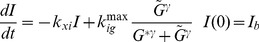
(6)where
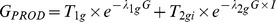
(7)and



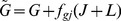
(8)From the steady state conditions it follows that:
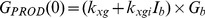
(9)

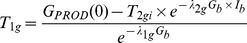
(10)

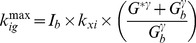
(11)


G and I represent glucose and insulin plasma concentrations, respectively. S, J and L are glucose amounts in stomach, jejunum and Ileum respectively, while R is a delay compartment necessary to describe the transit of the glucose load through the intestinal lumen.

#### Gastric emptying and gastrointestinal tract representation

The above mathematical representation (eq.1 to eq. 4) is a simplification of the glucose transit in the intestinal tract as described by Salinari et al. [13], where the progression of the glucose bolus was represented by means of a continuous one-dimensional process in which all glucose particles are transported from the proximal to the distal end of the small intestine with constant speed. Stomach glucose dynamics is described by equation 1, where the initial condition is the administered glucose dose *D*. The elimination (emptying) term depends on glucose amount in the stomach, where *k_js_* is the glucose transfer rate from stomach to jejunum. From the jejunum, glucose passes into the ileum through a delay compartment, and is absorbed with two potentially different constant rates of transfer (*k_gj_* and *k_gl_*). Equation 2 represents the variation of glucose amount in the jejunal compartment. The first term on the right hand side is glucose entry, which coincides with emptying from the stomach. The second term represents glucose absorption rate from the jejunum, which constitutes part of the rate of appearance into the glucose plasma compartment; the third term represents instead the amount of glucose not absorbed in the proximal intestinal tract, which transits to the Ileum (represented by equation 4) through the delay compartment R (represented by equation 3). The structure of equations 3 and 4 is clearly similar to that of equation 2.

#### Glucose dynamics

Equation 5 describes glucose dynamics, with the first term on the right hand side representing spontaneous glucose elimination rate and with the second term representing glucose tissue uptake due to insulin effect. The parameter *k_xgi_*, which is the insulin-dependent glucose elimination rate, represents an index of insulin sensitivity. Following identical considerations as those described elsewhere for the Single Delay Model (SDM) of the IVGTT [17], *k_xgi_* has the same dimensions and meaning as the insulin sensitivity index S_I_ computed from the “Minimal Model” [14].

Hepatic Glucose Production: the term *G_PROD_* (equation 7) represents Hepatic Glucose Output (HGO) as dependent on circulating plasma glucose and insulin. Liver glucose production is suppressed and glycogen-synthesis is enhanced in the presence of high plasma glucose and insulin concentrations. In the present formulation this indirect relationship has been represented by a decreasing exponential net glucose production for increasing glycemia and insulinemia. The first term in the *G_PROD_* equation describes net HGO as only dependent on plasma glucose levels, the second term instead takes into account the response of the liver also in the presence of high insulin concentrations.

Glucose Rate of Appearance: the last term of equation 5 represents glucose appearance into plasma, due to the administered dose *D*, which goes through the stomach and is absorbed from the small bowel. Since not all of the administered glucose amount is effectively absorbed, this term is multiplied by a fraction of absorption *f*. Delayed insulin action on glucose uptake has not been included because, even if it is widely believed that insulin action is in fact delayed [19], diffusion time of insulin into the interstitium is relatively fast, given the short recirculation time (approximately 2 minutes in the human). Further, previous experiences with an IVGTT model [17] showed that this term is not necessary to provide good adaptation to data. In that work, it was also shown that appreciable delay in the apparent effect of insulin was well reproduced by the model even in the absence of an explicit delay in the action of the hormone on target cells.

#### Insulin dynamics

Equation 6 describes insulin dynamics. The first term on the right-hand side represents insulin elimination, where *k_xi_* is the apparent first order insulin elimination rate; the second term describes insulin secretion due to both direct glucose stimulation and incretin hormones (equation 8).

Pancreatic Insulin Release: pancreatic insulin release is assumed to vary according to a Hill dynamics whose slope depends on the value of the exponent *γ*, and which reaches a maximal rate of release equal to 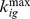. The coefficient *γ* represents the rate of attainment of maximal insulin secretion rate as glycemia and incretin hormones increase. Equations 9, 10 and 11 derive from equilibrium conditions of equations 5 and 6.

The meaning of each model parameter and the corresponding units of measurement are reported in Table 1.

**Table 1:** SIMO model parameter description.

Parameter	Unit of measurements	Description
*G_b_*	[mM]	basal plasma glucose concentration immediately before glucose administration
*I_b_*	[pM]	basal plasma insulin concentration immediately before glucose administration
*V*	[L/Kg]	glucose distribution volume
*D*	[mmol]	dose of glucose administered
*k_js_*	[min^−1^]	glucose transfer rate from stomach to jejunum
*k_gj_*	[min^−1^]	glucose transfer rate from jejunum to plasma
*k_rj_*	[min^−1^]	glucose transfer rate from jejunum to the delay compartment
*k_lr_*	[min^−1^]	glucose transfer rate from the delay compartment to ileum
*k_gl_*	[min^−1^]	glucose transfer rate from ileum to plasma
*k_xg_*	[min^−1^]	insulin independent first order glucose elimination rate
*k_xgi_*	[min^−1^ pM^−1^]	insulin dependent second order glucose elimination rate
*T_1g_*	[min^−1^ mM]	maximal rate of liver glucose production (in plasma concentration units) as dependent only on glycemia
*T_2gi_*	[min^−1^ mM]	maximal rate of liver glucose (in plasma concentration units) production as dependent on both glycemia and insulinemia
*λ_1g_*	[mM^−1^]	rate of decay of liver glucose production with increasing glycemia
*λ_2g_*	[mM^−1^ pM^−1^]	rate of decay of liver glucose production with increasing glycemia and insulinemia
*f*	[#]	fraction of bioavailable glucose from gastrointestinal tract
*k_xi_*	[min^−1^]	first order insulin elimination rate
*k_ig_^max^*	[min^−1^ pM]	maximal rate of insulin release
*γ*	[#]	pancreatic insulin secretion acceleration
*f_gj_*	[mM/mmol]	glucose-concentration equivalent effect of incretins on insulin release depending on gut glucose content.

### Empirical Glucose Homeostasis Descriptors

Besides model parameter estimates, for each subject a number of surrogate indices of glucose/insulin homeostasis were also computed (for a review see [20]): the Homeostasis Model Assessment (HOMA-IS), the Insulin Sensitivity Index Composite (ISIcomp) and the glucose Metabolic Clearance Rate (MCR_est_) were used to provide estimates of insulin sensitivity. A comparison was also made with the OGIS index [6] and with the IS_BREDA_ index [9], which are both model-inspired. Moreover a naif index (IS_NAIF_), computed simply as the inverse of the mean of the observed glycemias during the OGTT, was compared with all the other considered indices. The HOMA-BCF, the ratio (AUCrig) of area under the insulin concentration curve to area under the glucose concentration curve and the Insulinogenic Index (IGenic) were used as estimates of beta-cell function.

### Models of Glucose-insulin Homeostasis

Both the Dalla Man [10] and the Salinari [13] models were compared with the SIMO as alternative models for the interpretation of the OGTT. In all three models the insulin sensitivity index SI appears as one of the parameters to be estimated.

### Patient Sample

After signing the written informed consent, a total of 118 subjects were admitted to the Outpatient Clinic of the Institute of Endocrinology in Prague. Women participated between the 1st and 5th day of their spontaneous menstrual cycle. The protocol was approved by the Ethical Committee of the Institute of Endocrinology in Prague (Czech Republic) and was conducted according to the principles of the Helsinki Declaration. Weight (to the nearest 0.1 kg) and height (to the nearest cm) were measured. Basal fasting blood samples were taken from the cubital vein. Subsequently, a 75 g glucose oral load was administered and additional blood samples were gathered at 30, 60, 90, 120, 150 and 180 min for measuring glucose and insulin concentration. Samples were centrifuged and serum was stored at −20°C until analysis. Insulin was assayed by IRMA (Immunotech, Prague, Czech Republic), with an intra- and inter-assay CV of 4.6 and 5.3%, respectively. Glucose concentration was measured using the glucoxidase method (Beckmann Glucose Analyser, Fullerton, CA) in venous plasma, with an intra- and inter-assay CV of 1.8 and 2.6%, respectively. Of the original 118 subjects, 78 had at least five valid measurements out of seven for glycemia and five out of seven for insulinemia, and were retained for model identification. According to fasting and 2 h glucose, subjects were divided into 5 different metabolic groups following the American Diabetes Association criteria [21].

### Statistical Parameter Estimation

Individual model parameters were obtained for each subject, by fitting the new proposed model to glucose and insulin plasma concentrations by Weighted Least Squares (WLS). Weights were obtained by multiplying the inverses of the squares of the expectations by the squared coefficients of variation, which were set at 22% for glucose and 31% for insulin, as estimated in a previous work [18] from a series of Glycemia and Insulinemia determinations in humans after an Intravenous Glucose Tolerance Test. It is to be noted in this context that the 22% and 31% CVs in-vivo observations are obviously much larger than the commonly reported CVs for repeated measurements on the same blood sample (e.g. 6% for insulin and 2% for glucose [22]). All observations on glucose and insulin were considered in the estimation procedure. Parameter estimation was also performed for the Salinari et al. [13] model as well as for the Dalla Man model [10] (a synthetic description of both models is reported in Appendix S1 and Appendix S2 respectively); for these two models only glucose data were used for the fitting procedure whereas interpolated insulin observations entered the models as a forcing function as suggested by the respective Authors. Due to the lack of GLP1 data, when applying the Salinari et. al. model some parameters were set at their average values, as reported in the original work [13] and as summarized in the following.

The original set of parameters of the Salinari et. al. [13] model to be estimated for each subject consisted in fact of seven parameters: *k*, *c_2_*, *S_G_*, *S_I_*, *p*, *GLP_b_*, and *b_GLP_* (see Appendix S1 for a more detailed description of the implemented model). The remaining model parameters were set to fixed values by the same authors. In particular, in the present work the values used for the fixed parameters are those reported in the legend of Figure 2 in [13], with *z_1_* = 0 cm, *z_2_* = 630 cm, *s_1_* = 35 cm, *s_2_* = 140 cm, *L = *630 cm, *u = *3.5 cm/min, *β = *1.2 and V = 0.19 L/kg. Parameters *GLP_b_* and *b_GLP_* should be estimated from the observed GLP-1 dynamics. Because of the lack of GLP-1 observations, in the present work *GLP_b_*, and *b_GLP_* were set to the average values reported in Table 1 of [13], that is to 17.6 pM and 6.33×10^−9^ L^−1^ respectively, while parameters *k*, *c_2_*, *S_G_*, *S_I_* and *p* were free and were therefore estimated.

**Figure 2:**
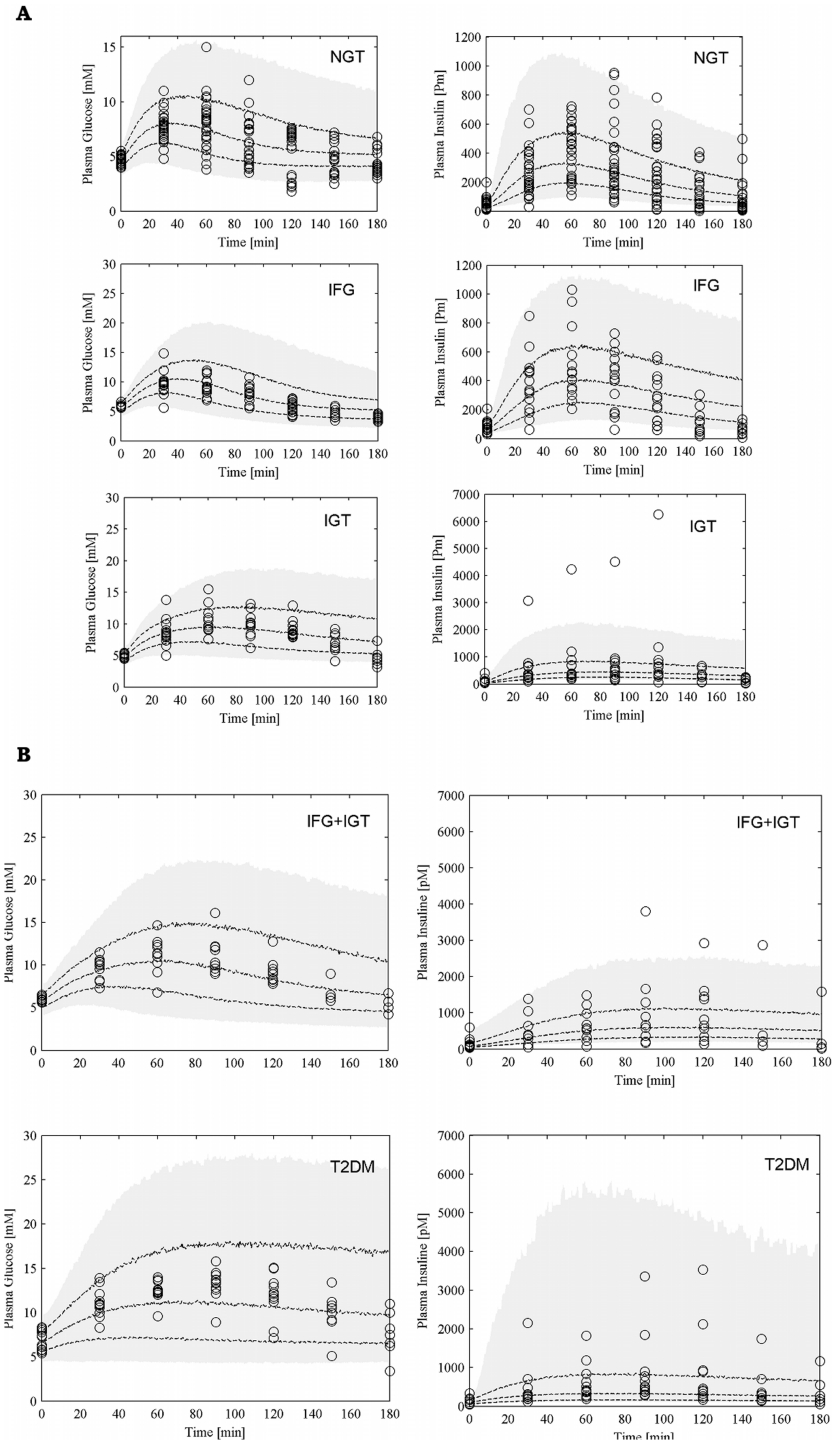
Visual Predictive Check. Visual Predictive Check (VPC) for each of the 5 groups (panel A for NGT, IFG and IGT; panel B for IFG+IGT and T2DM). For each patient 200 simulations were performed with the model: the shaded area represents the 90% prediction interval, dashed lines represent the 25-th, 50-th and 75-th percentile. Observed data are reported as circles.

Due to evident *a priori* unidentifiability, some parameters were kept fixed throughout the optimization process. The distribution volume *V_g_* was set at 0.19 liters per kg body weight, the standard assessment of glucose distribution space [23]. Glucose effectiveness *k_xg_* was set at 0.001 min^−1^: in this case, previous work [17] showed that this coefficient was not necessary for a good fit to IVGTT data; also, the mass of glucose-metabolizing tissue, other than the brain (assumed having constant glucose consumption), is insulin-dependent (muscle and adipose tissue); on the other hand, some linear glucose consumption could be attributed to red blood cells and possibly other tissues, and therefore a small but nonzero value for *k_xg_* was selected. The coefficient *k_gj_* was set at 0.042 in order to have, empirically, a residue of 8% of an intra-jejunally administered bolus after 1 hour, a number which was felt plausible by the medical doctors with whom the authors collaborate. Similarly, the coefficient *f_gj_* was set at 0.02 in order to express the empirical idea that an addition of 50 mmol (9 g, approx. two teaspoons) of glucose in the bowel may have the same stimulating effect (via the incretin mechanism) on pancreatic insulin release as the addition of 1 mM to plasma glucose concentration. The coefficients *k_rj_* and *k_lr_* were empirically set at 0.09 min^−1^ and at 0.06 min^−1^ respectively in order for essentially 100% of jejunal glucose content to reach the ileum within 3 hours. For all of the above parameter values, the concern was to acceptably assess at least the order of magnitude involved, leaving to further investigation the issue of obtaining more precise individual estimates for a given patient.

A Nelder-Mead simplex algorithm was used for all optimizations [24].

For each subject, the estimate of the approximate covariance matrix of the parameter estimates was computed according to asymptotic distribution theory. Given the independency of errors and given the hypothesized structure of the covariance of errors, the estimated parameter vector 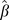 is normally distributed with mean 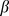, consisting of the true values of the model parameter vector, and covariance matrix equal to:
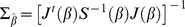
whose estimate is obtained by replacing 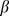 with 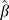, and where *S* is the covariance matrix of the error vector.

Comparisons among groups on the estimated model parameters as well as on the empirical glucose homeostasis indices were performed by means of one-way Analysis of Variance. Post-hoc multiple comparisons were performed using the Least Significant Difference (LSD) test with the Hochberg correction. A P-value<0.05 was assumed to be statistically significant.

### Model Validation

A Visual Predictive Check (VPC) was performed for the SIMO model in order to have a visual inspection of its predictive properties. Parameter estimation was conducted in terms of logarithms; at the second stage of the statistical model formalization a normal distribution of the logarithm of the parameters was hypothesized. In particular we hypothesized the presence of five sub-populations (indexed by *g*), one for each different glucose tolerance group. Formally:
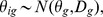
where *θ_ig_* is the individual parameter, in logarithm form, of subject *i* belonging to sub-population *g*, *θ_g_* and *D_g_* are the true sub-population mean and variance-covariance matrix respectively. In a two-stage parameter estimation method the estimates of *θ_g_* and *D_g_* are given by the sample mean and covariance of the estimates of *θ_ig_*.

For each of the 5 groups the sample mean and the sample variance-covariance matrix were then used to draw parameter samples from a multivariate normal distribution. Measurement errors were hypothesized to be independent and normally distributed with zero mean and variance equal to the squares of the expectations times the squared coefficients of variation, set at 22% for glucose and 31% for insulin observations, as stated in the previous subsection. For each patient in the five groups 200 simulations were performed with the model, using parameters drawn from the corresponding distribution and observation errors drawn from the relative error distribution. The 25^th^, 50^th^ and 75^th^ percentile were computed and plotted along with the observed data. The 90% prediction interval (from the 5^th^ percentile to the 95^th^ percentile) was also reported as shaded area.

## Results

In the following, the three models compared have been indicated as SIMO model (Simple Interdependent glucose/insulin MOdel) to refer to the new proposed model, COMO (COntinuous GI tract MOdel) for the Salinari et. al. model, and DMMO for the Dalla Man MOdel.

The three models have been fitted to individual OGTT experiments from 78 subjects belonging to 5 groups with different types and degrees of glucose metabolism impairment.

### Sample Description

Table 2 reports the anthropometric characteristics of the analyzed subjects, by group. Age was significantly different among groups (P<0.001 by ANOVA), with the NGT group presenting a lower average age with respect to all the other groups. BMI also increased with worsening of the metabolic status: post-hoc comparisons showed that patients with T2DM and with IGT plus IFG have a greater BMI than patients with only IGT or IFG. NGT subjects had the lowest BMI. ANOVA on basal glucose (G_b_) and basal insulin (I_b_) plasma concentrations resulted significant overall (P<0.001 for both variables). Post-hoc analyses highlighted no significant difference between IFG and IGT+IFG and between IGT and NGT for basal glycemia. Basal insulinemia was significantly different between IFG+IGT and all the other groups apart from T2DM; T2DM subjects had higher basal insulinemia than NGT’s subjects. Table 3 reports the computed empirical indices of glucose/insulin homeostasis by group. Glucose levels at 2 hours (G_2h_) were higher in T2DM, IFG+IGT and in IGT subjects (11.95±2.43, 9.22±1.43 and 9.17±1.37 respectively, P<0.001 from ANOVA). Average values of G_2h_ in the other two groups were 5.71±2.13 in NGT patients and 5.88±1.08 in the IFG group.

**Table 2:** Anthropometric characteristics of the subjects: means and standard deviations by group.

	NGT N = 28		IFG N = 15		IGT N = 13		IFG+IGT N = 10		T2DM N = 12	
	mean	SD	mean	SD	mean	SD	mean	SD	mean	SD
**Age [year]**	35.35	13.65	48.25	12.62	47.74	11.49	49.56	14.72	52.00	12.06
**Weight [Kg]**	74.98	15.92	80.55	11.56	83.45	17.01	89.98	20.99	83.28	19.81
**Height [cm]**	168.8	9.3	171.5	8.2	170.4	13.2	169.0	9.5	163.8	10.20
**BMI [kg/m^∧^2]**	26.33	5.52	27.32	3.18	28.71	4.73	31.00	5.00	30.88	5.88
**Basal glucose [mg/dl]**	85.54	6.71	106.04	5.14	90.26	5.70	106.90	5.96	123.03	19.95
**Basal insulin [mU/L]**	7.29	5.32	11.52	6.50	14.08	14.90	23.01	24.28	18.66	12.17
**Gender F/M (%F/%M)**	18/10 (64.29/35.71)		7/8 (46.67/53.33)		7/6 (53.85/46.15)		7/3 (70.00/30.00)		10/2 (83.33/16.67)	

**Table 3:** Empirical indices of glucose/insulin homeostasis by group.

	NGT		IFG		IGT		IFG+IGT		T2DM		P
	mean	SD	mean	SD	mean	SD	mean	SD	mean	SD	
**HOMA-IS**	1.07	0.95	0.43	0.22	0.56	0.34	0.30	0.20	0.25	0.14	<0.001
**ISIcomp**	9.20	6.58	4.54	2.33	4.34	2.43	3.02	2.63	2.40	1.24	<0.001
**MCRest**	9.65	1.72	9.14	1.17	7.66	1.96	6.70	2.23	6.24	1.90	<0.001
**OGIS**	622.4	175.22	541.3	85.58	469.9	121.98	375.2	110.63	371.5	102.71	<0.001
**IS_BREDA_**	20.70	17.91	11.59	7.02	6.24	5.12	6.87	8.16	4.16	3.94	<0.001
**IS_NAIF_**	0.17	0.042	0.14	0.014	0.12	0.016	0.11	0.014	0.09	0.012	<0.001
**HomaBCF**	6.46	4.00	5.50	3.42	9.86	8.48	10.11	9.28	7.26	6.16	0.19
**AUCrig**	41.26	19.75	46.27	22.62	68.39	72.64	73.35	57.65	52.17	47.72	0.19
**IGenic**	92.53	64.23	80.94	45.46	228.10	449.62	88.95	63.11	71.07	65.78	0.20
**AUCG**	1043.86	268.08	1167.32	183.92	1363.56	274.45	1348.32	258.38	1781.38	307.48	<0.001
**AUCI**	41851.50	20816.66	53991.00	26365.69	96569.31	113092.57	106146.60	105779.98	91675.50	82809.23	0.03
**G2h**	5.71	2.13	5.88	1.08	9.17	1.37	9.22	1.43	11.95	2.43	<0.001

### Validation of the Model

The predictive properties of the SIMO model were assessed by performing a visual predictive check (VPC) for each of the 5 groups. For each patient 200 simulations were performed with the model and Panels A and B of Figure 2 report the 90% prediction interval (shaded area), the 25^th^, 50^th^ and 75^th^ percentile (dashed lines) as well as all the observed data from patients in the considered group. In interpreting the figures, it must be noticed that scale varies after NGT for glycemia and after IFG for insulinemia.

**Figure 3:**
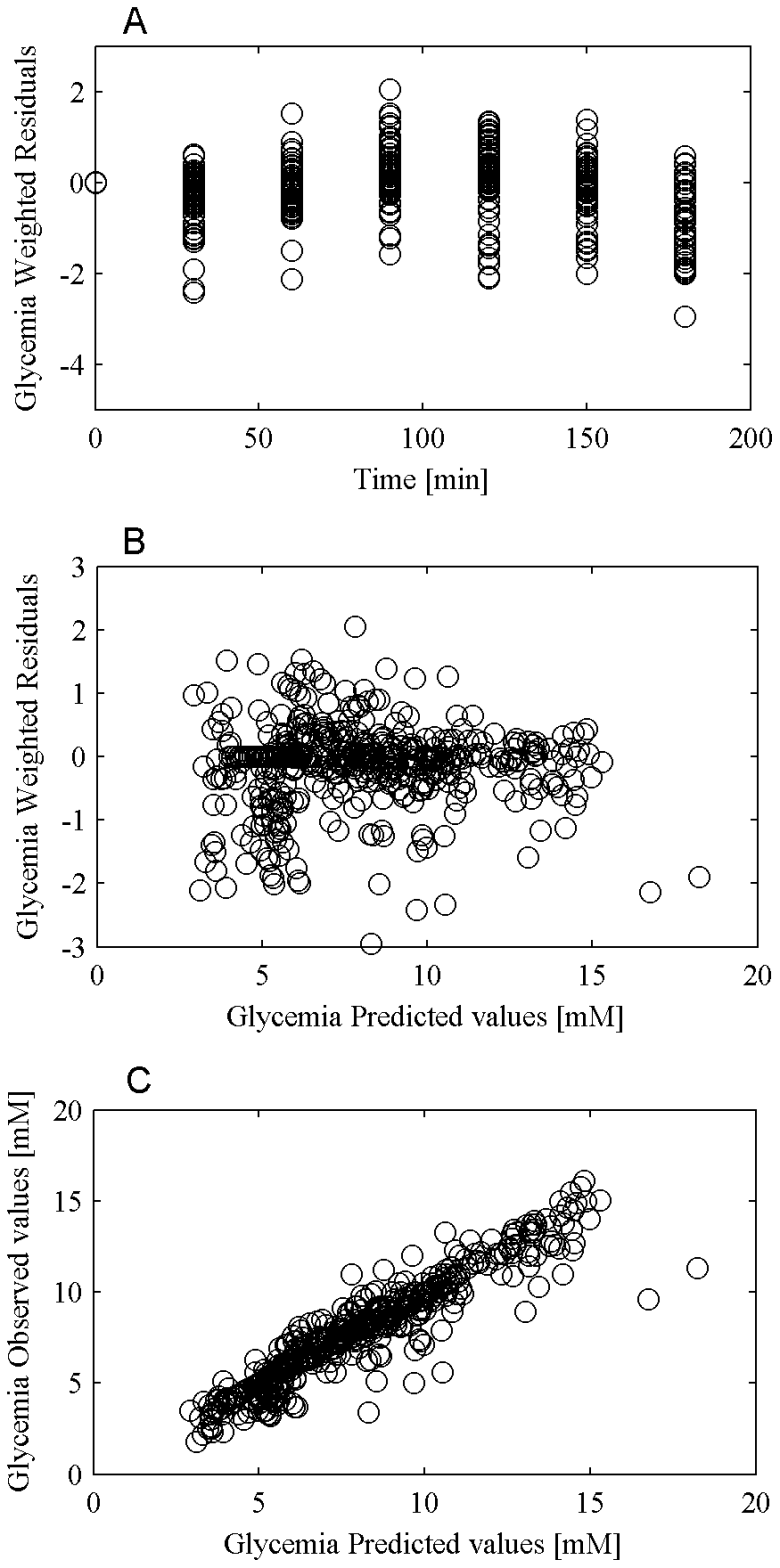
Glucose prediction diagnostic plot. Panel A reports weighted residuals versus time, panel B reports weighted residuals versus glucose predictions and panel C reports observed concentrations versus predicted concentrations.

**Figure 4:**
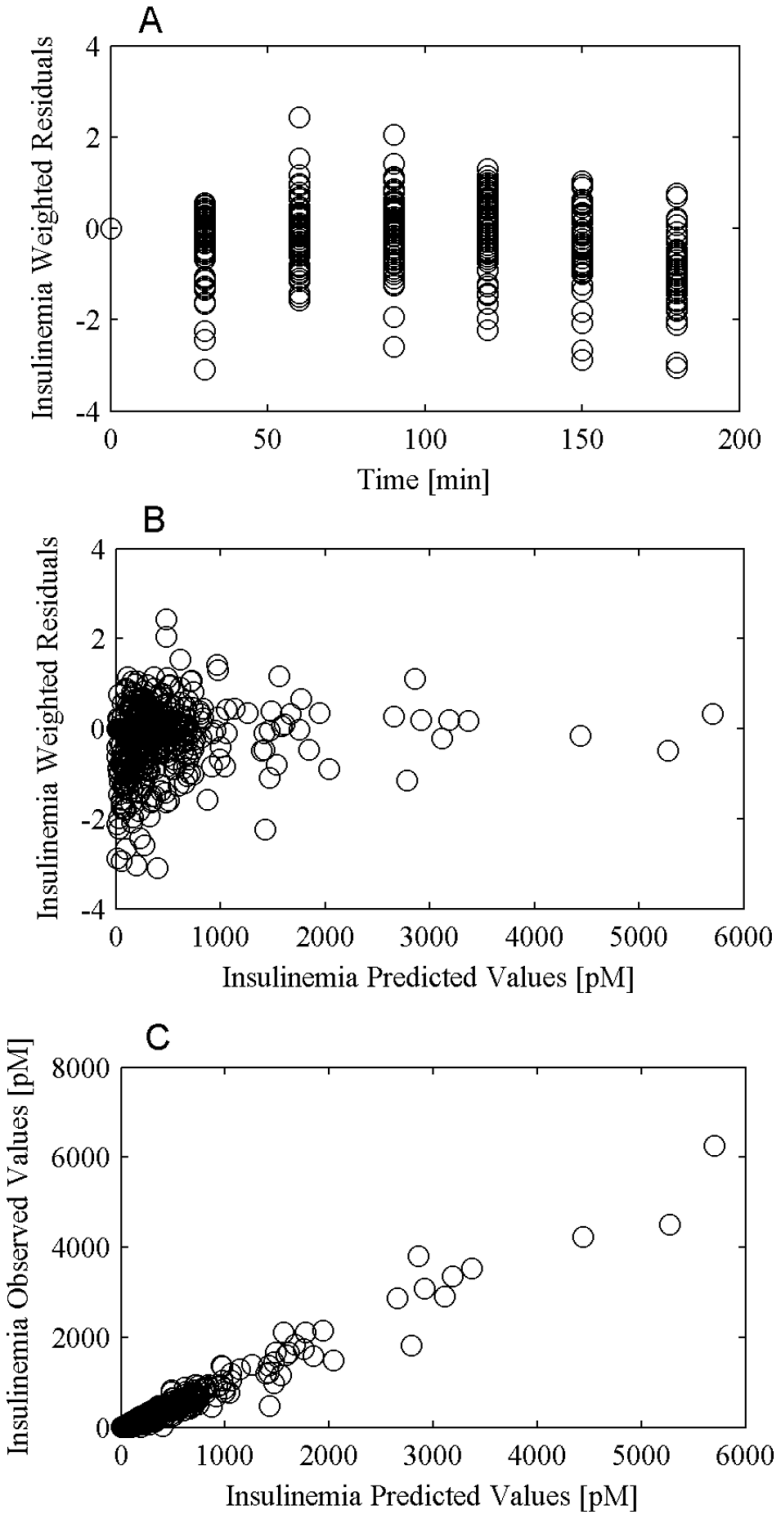
Insulin prediction diagnostic plot. Panel A reports weighted residuals versus time, panel B reports weighted residuals versus insulin predictions and panel C reports observed concentrations versus predicted concentrations.

The performance of the model in predicting glycemia and insulinemia observations and the validity of the assumptions made in relation to the error variance were evaluated by means of the diagnostic plots of weighted residuals versus time, weighted residuals versus predicted concentrations and observed concentrations versus predicted concentrations. Figures 3 and 4 from panel A to C report the three plots for glycemia and insulinemia respectively.

### Model Insulin Sensitivity Indices: a Comparison among Models

Comparison among models was made with particular attention to the results related to insulin sensitivity indices: IS_COMO_, IS_DMMO_ and *k_xgi_* (the insulin sensitivity parameter from the SIMO).

The values of G_2h_ in the investigated groups were highly and inversely correlated with the insulin sensitivity parameters *k_xgi_* (r = −0.57, P<0.001) and IS_DMMO_ (r = −0.34, P = 0.003). No correlation was observed between G_2h_ and IS_COMO._

Correlations of model-derived and empirical sensitivity indices are reported in Table 4. The *k_xgi_* index correlates better than the other model derived index SI_DMMO_ with all other insulin sensitivity indices. IS_BREDA_ appears to have the highest coefficients of correlation with all other indices, however, it does not discriminate between the different glucose tolerance groups: for it, LDS contrasts only separate NGT from all other groups (Table 4). The IS_NAIF_ index was also highly correlated with all other indices and was moreover able to discriminate all groups from each other.

**Table 4:** Correlations among empirical measures of insulin sensitivity and model-derived insulin sensitivity indices.

	HOMA-IS	ISIcomp	MCRrest	OGIS	IS_BREDA_	IS_NAIF_	IS_DMMO_	IS_COMO_	k_xgi_
**HOMA-IS**	1	0.87**	0.50**	0.54**	0.56 **	0.45**	0.31*	0.016 NS	0.49**
**ISIcomp**	0.87**	1	0.60**	0.77**	0.87**	0.71**	0.62**	0.02 NS	0.79**
**MCRrest**	0.50**	0.60**	1	0.69**	0.65**	0.71**	0.33*	0.084 NS	0.56**
**OGIS**	0.54**	0.77**	0.69**	1	0.75**	0.83**	0.53**	0.080 NS	0.72**
**IS_BREDA_**	0.56**	0.87**	0.65**	0.75**	1	0.81**	0.77**	−0.02 NS	0.90**
**IS_NAIF_**	0.45**	0.71**	0.71**	0.83**	0.81**	1	0.54**	*0.015 NS*	0.72**
**IS_DMMO_**	0.28*	0.51**	0.33*	0.48**	0.69**	0.58**	1	−0.07 NS	0.63**
**IS_COMO_**	*0.016 NS*	*0.02 NS*	*0.084 NS*	0.080**	−*0.02 NS*	*0.015 NS*	−*0.07 NS*	1	−0.05 NS
**k_xgi_**	0.49**	0.79**	0.56**	0.72**	0.90**	0.72**	0.70**	−*0.05 NS*	1

Asterisks indicate significance of the correlations: * P<0.01, **P<0.001, NS Not Significant.

**HOMA-IS:** Homeostasis Model Assessment.

**ISIcomp:** Insulin Sensitivity Index Composite.

**MCRrest**: glucose Metabolic Clearance Rate.

**OGIS**: Oral Glucose Insulin Sensitivity index as estimated in [6].

**IS_BREDA_**: Insulin Sensitivity index as derived in [9].

**IS_NAIF_**: Insulin Sensitivity index computed as the inverse of the mean of the observed glycemias during the OGTT.

**IS_DMMO_:** Insulin Sensitivity index as derived from the Dalla Man model [10].

**IS_COMO_:** Insulin Sensitivity index as derived from the Salinari model [13].

**k_xgi_:** Insulin Sensitivity index as derived from the proposed SIMO model.

ANOVA on *k_xgi_* values across groups resulted significant overall (P<0.001), and post-hoc comparisons highlighted the separation between three different groups: NGT patients presenting with the highest value (8.62×10^−5^±9.36×10^−5^), followed by IFG patients (5.30×10^−5^±5.18×10^−5^) and with the combined IGT, IFG+IGT and T2DM patients presenting with the lowest average values (2.09×10^−5^±1.95×10^−5^, 2.38×10^−5^±2.28×10^−5^ and 2.38×10^−5^±2.09×10^−5^ respectively). Table 5 reports single group mean values as well as the average over the aggregated groups. No significance was obtained when comparing the other two model-derived insulin sensitivity indices across groups.

**Table 5:** Insulin sensitivity indices estimates by group.

k_xgi_ [min^−1^ pM^−1^]	NGT	IFG	T2DM	IFG+IGT	IGT
Mean	8.62E-05	5.30E-05	2.38E-05	2.38E-05	2.09E-05
**Standard Deviation**	9.36E-05	5.18E-05	2.09E-05	2.28E-05	1.95E-05
**group mean** ***±SD***			***2.27E-05±2.04E-05***		
**k_xgi_ trimmed** [min^−1^ pM^−1^]	**NGT**	**IFG**	**T2DM**	**IFG+IGT**	**IGT**
**Mean**	5.70E-05	4.12E-05	2.00E-05	1.90E-05	1.69E-05
**SD**	2.70E-05	1.70E-05	8.18E-06	1.29E-05	6.04E-06
**group mean** ***±*** **SD**	***5.13E-05±2.47E-05***		***1.86E-05±8.86E-06***		
**k_xgi_ interpolated** [min^−1^ pM^−1^]	**NGT**	**IFG**	**IFG-IGT**	**IGT**	**T2DM**
**Mean**	7.10E-05	5.88E-05	2.61E-05	2.45E-05	1.91E-05
**Standard Deviation**	6.98E-05	5.37E-05	2.23E-05	1.58E-05	1.39E-05
**group mean** ***±*** **SD**			***2.31E-05±1.71E-05***		
**k_xgi_ interpolated trimmed** [min^−1^ pM^−1^]	**NGT**	**IFG**	**IGT**	**IFG+IGT**	**T2DM**
**Mean**	5.35E-05	4.20E-05	2.13E-05	1.95E-05	1.78E-05
**Standard Deviation**	2.05E-05	1.54E-05	5.69E-06	1.48E-05	5.66E-06
**group mean±SD**	***4.94E-05±1.93E-05***		***1.96E-05±9.46E-6***		
**IS_COMO_** [min^−1^ pM^−1^]	**IFG**	**IGT**	**NGT**	**IFG+IGT**	**T2DM**
**Mean**	1.06E-02	6.07E-03	3.31E-04	2.32E-04	2.71E-05
**Standard Deviation**	2.66E-02	1.85E-02	1.14E-03	5.47E-04	5.83E-05
**IS_COMO_ trimmed** [min^−1^ pM^−1^]	**IFG**	**IGT**	**NGT**	**IFG+IGT**	**T2DM**
**Mean**	4.17E-04	9.04E-05	8.94E-05	5.42E-05	2.78E-06
**Standard Deviation**	9.84E-04	5.00E-05	5.35E-05	4.88E-05	6.25E-06
**IS_DMMO_** [min^−1^ pM^−1^]	**NGT**	**IFG**	**T2DM**	**IGT**	**IFG-IGT**
**Mean**	3.91E-03	2.27E-03	1.32E-03	8.96E-04	3.62E-04
**Standard Deviation**	9.68E-03	4.49E-03	2.16E-03	1.99E-03	9.40E-04
**IS_DMMO_ trimmed** [min^−1^ pM^−1^]	**T2DM**	**NGT**	**IFG**	**IGT**	**IFG-IGT**
**Mean**	6.59E-04	3.80E-04	2.59E-04	2.58E-04	6.54E-05
**Standard Deviation**	7.99E-04	4.90E-04	1.93E-04	4.75E-04	7.65E-05

**IS_DMMO_:** Insulin Sensitivity index as derived from the Dalla Man model [10].

**IS_COMO_:** Insulin Sensitivity index as derived from the Salinari model [13].

**k_xgi_:** Insulin Sensitivity index as derived from the proposed SIMO model.

The term “**trimmed**” was used to identify the subgroup consisting of values of the insulin sensitivity index between the 20th and 80th percentile.

The term “**interpolated**” coupled with the k_xgi_ index refers to parameter estimate values obtained when observed insulin concentrations are interpolated rather than fitted.

Among model-based insulin sensitivity indices, *k_xgi_* presented the lowest sample average coefficient of variation over the five groups (25.43%), with the average CVs for IS_COMO_ and IS_DMMO_ being equal to 70.32% and 57.75% respectively. In order to attempt to limit the impact of outlier estimates, which could obscure the physiologic significance of the indices, comparisons among groups in terms of insulin sensitivity were also repeated when considering only values between the 20^th^ and 80^th^ percentiles. Results for this “trimmed” subgroup are also reported in Table 5. While ANOVA was again non-significant for both SI_DMMO_ and SI_COMO_, for *k_xgi_* ANOVA was significant and post-hoc analysis isolated two (instead of three) groups: NGT and IFG together versus IGT, IFG+IGT and T2DM together.

The comparison in terms of dispersion of the parameter estimates was performed only between SIMO and DMMO models, since COMO model performs poorly also in terms of insulin sensitivity estimation. This apparently poor performance of the COMO model is essentially due to a large number of model parameters, which, in the absence of GLP-1 observations, have to be fixed to some average or plausible values.

Keeping in mind that the results are valid only when asymptotic theory holds, summary results related to the computations of the asymptotic dispersion around the parameter estimates are reported in Tables S1, S2 and S3 in the Supporting Information. Inversion of the matrix 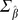 was possible only in 14 case out 78 for the DMMO model whereas for the SIMO model the covariance matrix could be computed for every subject. Table S1 reports the median of the coefficients of variation for each free parameter of the SIMO model, estimated on logarithmic scale. Table S2 reports results obtained for all free parameters of the DMMO model, as well as for the SI_DMMO_ index, whose dispersion was computed from the dispersion of parameters *p_2_* and *p_3_*, on which the insulin sensitivity index SI_DMMO_ depends. From Table S1 it can be seen that the parameter *k_xgi_* is the parameter whose estimate dispersion is the lowest in all five groups. The other parameters which are estimated with good precision are the parameters *k_xi_*, *γ* and *k_js_*.

From Table S2 it can be seen that, over the 14 subjects which the DMMO model estimated with sufficient numerical stability to allow the computation of the asymptotic variance-covariance matrix, the best precision is obtained for the SI_DMMO_ and p_3_ parameters in all groups. Table S3 presents a direct comparison between the SIMO and DMMO models in terms of precision of the respective Insulin Sensitivity index estimates. The first column on the left reports results obtained for the SIMO *k_xgi_* index over the whole sample of 78 subjects, the column in the center reports results for the SIMO *k_xgi_* over the best 14 patients in terms of estimate precision and the column on the right reports results for SI_DMMO_ over the subsample of 14 subjects for whom the covariance matrix of the estimates could be computed. The table reports the average estimates, in natural units, the median of the coefficients of variation and their Interquartile Range.

The results obtained with the SIMO model over the whole sample are comparable with those obtained for the best subsample, and much better than the results obtained with the DMMO over the 14 subjects for which a Hessian could be computed and inverted: not only the coefficients of variation are smaller, hence the estimates are more precise, but the average values of insulin sensitivity obtained with the DMMO model average more than 200×10^−5^ for NGT subjects (more than 20 times the “normal” value which should be of the order of 10^−4^ [14]), 22 and 35×10^−5^ respectively for IFG’s and T2DM’s, which would imply that T2DM subjects are 6 to 7 times more insulin sensitive than IGT’s and IFG+IGT’s. These results are not plausible and are clearly due to numerical instability.

**Figure 5:**
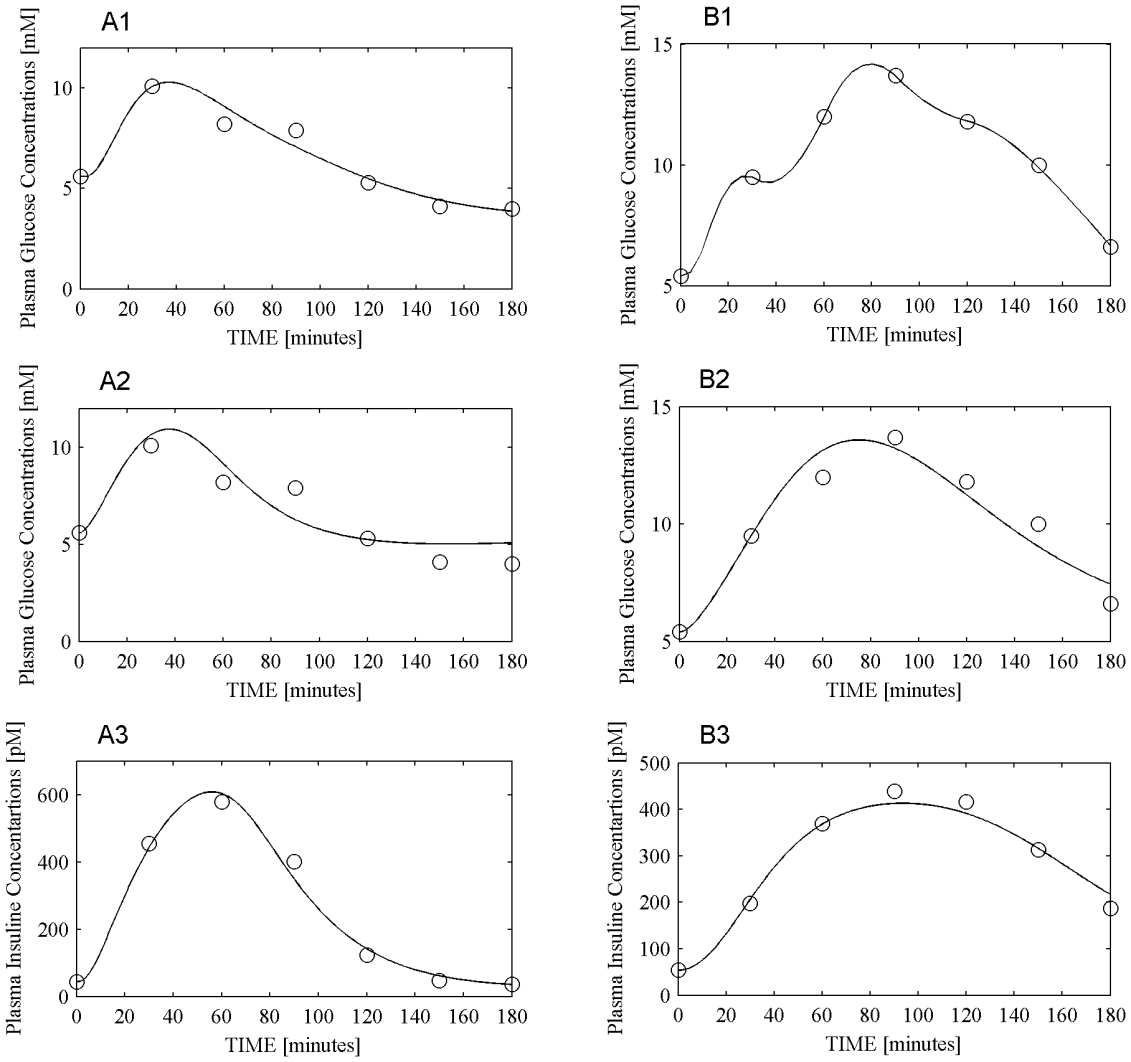
COMO, SIMO and DMMO model data fitting. Panels A reports glucose and insulin dynamics for one IFG patient. Panel A1 reports observed (circles) plasma glucose concentrations together with their prediction using the COMO model (continuous line). Panels A2 and A3 report respectively glycemia (A2) and insulinemia (A3) concentrations (circles), together with the corresponding predictions obtained with the SIMO model (continuous line). Panels B report glucose and insulin dynamics for one T2DM patient. Panel B1 reports observed (circles) plasma glucose concentrations together with their prediction using the DMMO model (continuous line). Panels B2 and B3 report respectively glycemia (B2) and insulinemia (B3) concentrations (circles), together with the corresponding predictions obtained with the SIMO model (continuous line).

In order to study model behavior in case of extreme parameter estimates, Figure 5 panel A1 reports the predicted and observed plasma glucose concentrations obtained with the COMO model for one IFG patient whose insulin sensitivity index IS_COMO_ was estimated at the low limit of optimization (10^−10^), while Figure 5 panel B1 reports the predicted and observed plasma glucose concentrations obtained with the DMMO model for one T2DM patient whose insulin sensitivity index IS_DMMO_ was estimated at 3.26×10^−3^ (very high). The OGTT data from these same two patients are reported again in Figure 5 panels A2–A3 (IFG patient) and panels B2–B3 (T2DM patient), together with curves obtained by fitting these subjects with the SIMO model.

**Figure 6:**
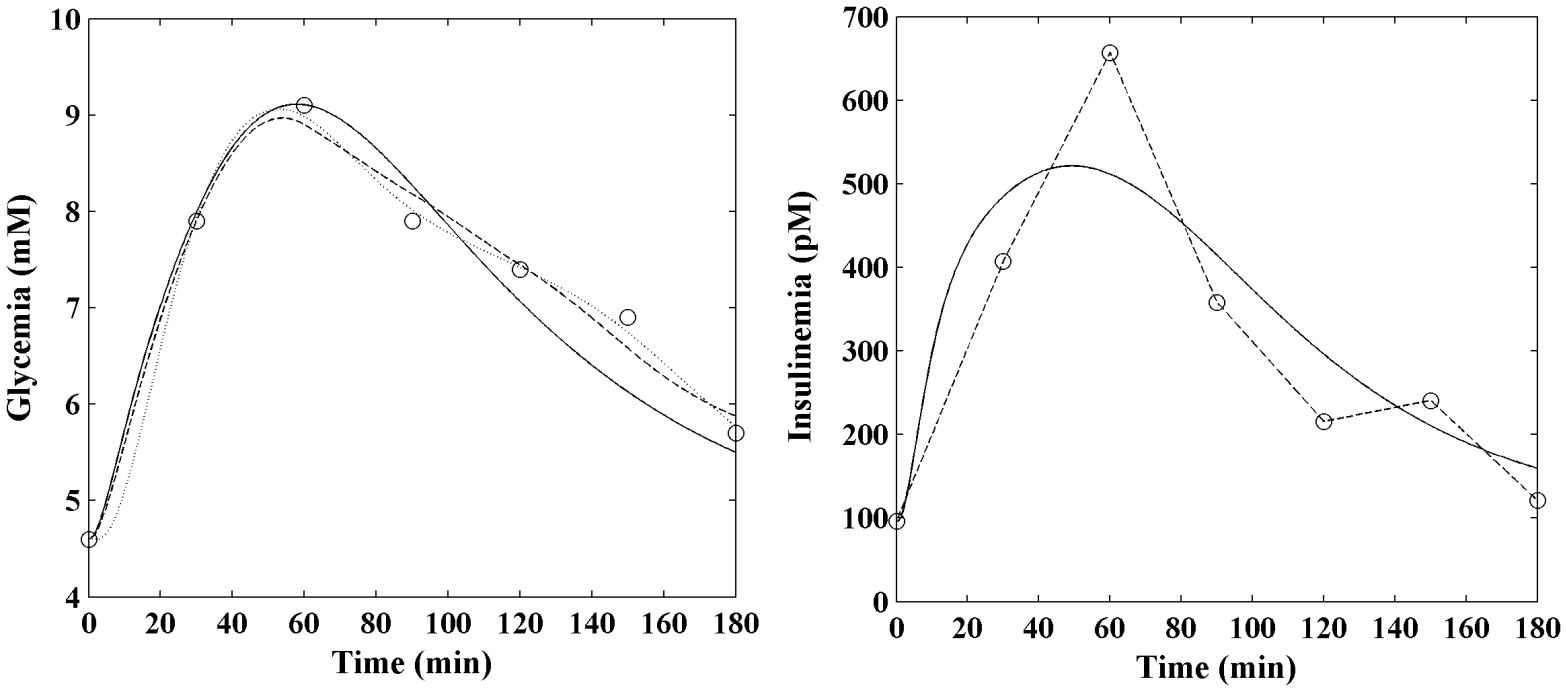
Comparison between fitting with predicted and interpolated insulin concentrations. Observed glucose and insulin data (circles) for one NGT patient. The COMO fitted model (dotted line) is shown together with the SIMO model either when insulin is fitted (solid black line) or when insulin is interpolated (dashed line).

The problem introduced by incorrectly using interpolated noisy observations as forcing function for model fit was explored, for the newly proposed model, by using interpolated insulin instead of predicted insulin. Figure 6 compares, for one NGT patient, the fitting performance of the COMO model (dotted line) with the SIMO model either when insulin is fitted (solid black line) or when insulin is interpolated (dashed line).

### Insulin Secretion

All empirical insulin secretion descriptors resulted non-significantly different among groups (Homa-BCF, P = 0.19; AUCrig, P = 0.19; IGenic, P = 0.20). The SIMO parameter 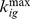, which represents maximal insulin secretion rate, also resulted non-significant (P = 0.31) even if subjects seemed to divide into two groups: IGT and IFG+IGT patients with larger 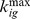 values (71.84±66.52 and 72.01±112.09 respectively), and NGT, IFG and T2DM patients with smaller 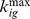 values (45.93±41.67, 34.21±31.72 and 43.6±30.93 respectively). The empirical insulin secretion descriptors were positively correlated with 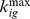 (r = 0.62, P<0.001; r = 0.41, P<0.001; r = 0.38, P<0.001 for correlation with Homa-BCF, AUCrig and IGenic respectively).

The parameter *γ* resulted significantly different among groups (overall P = 0.009 from ANOVA); post-hoc comparisons highlighted the presence of three groups: T2DM patients presenting with the highest value at 7.98±5.51, IFG and IFG+IGT together for which parameter values were 6.58±3.69 and 6.21±4.76 respectively and NGT and IGT patients together with values equal to 4.42±0.96 and 4.15±0.81 respectively.

### Liver Glucose Output

Even if the rate of decay of liver glucose production with increasing glycemia, *λ_1g_*, did not result significantly different among groups (P = 0.07 from ANOVA), it does show a trend, with the lowest values for IFG subjects (0.39±0.42) and the largest value for the IGT group (0.80±0.26); values for NGT, IFG+IGT and T2DM were 0.58±0.39, 0.60±0.33 and 0.58±0.30 respectively.

Tables S4, S5 and S6 report descriptive statistics for free and determined model parameters, by clinical group, for the SIMO, DMMO and COMO models respectively.

## Discussion

Diseases associated with glucose intolerance are widespread and diagnosis is fundamental for prevention and treatment. Therefore, the need for a low cost, easily and widely applicable tool for insulin sensitivity detection is unquestionable. Mathematical models can allow the computation of metabolic indices from glucose tolerance tests and can offer a real, practical opportunity to quickly diagnose patient status: their usefulness is enhanced if they can reliably provide at the same time measures of insulin secretion together with measures of insulin sensitivity.

In the present work a Simple Interdependent glucose/insulin MOdel, SIMO, is proposed and used to fit data from a cohort of subjects with different types and degrees of glucose metabolism impairment, ranging from normal (NGT) to impaired (IGT, IFG, IFG+IGT) glucose tolerance, to Type 2 Diabetes Mellitus (T2DM). The present model exhibits some specific features: the first one is the incorporation of an explicit incretin term; the second one is the derivation of the glucose rate of appearance; the third one is the mathematical representation of insulin release; the fourth one is a consistent representation of the dynamics of hepatic glucose output.

### Modeling the Incretin Effect

In the present work the incretin effect was modeled as a nonlinear function of the content of glucose in the gastrointestinal tract, expressing the effect of gastrointestinal hormones on glucose-stimulated insulin secretion. The importance of considering gut hormones in humans is supported by different studies that have demonstrated that incretins account for about 50–70% of the insulin response to oral glucose [25]. While in most of hitherto published models the direct effect of incretins was not taken into account, recently some works inserted incretin terms into models for OGTT data [26], [27]. In Silber et al. [26] the incretin effect was introduced as a linear function of glucose absorption rate; in Brubaker’s work [27] variation over time of GLP-1 and GIP gastrointestinal hormones was modeled by means of an ordinary differential equation and the incretin effect was directly considered as a first order effect on insulin production. Besides the fact that, as stated by these last Authors, the “model’s insulin response over an extended time course falls short of that which is observed experimentally”, the work was completely simulative, no parameter estimation was performed and parameter values as well as some functional forms were derived from published data. In Salinari et al. [13] modeling of the release of gastrointestinal hormones was used to validate the modeling of the time course of the rate of appearance of glucose, which represented the focus of that work, and was not used to understand the mechanisms underlying insulin secretion: plasma insulin observations were in fact interpolated and used as forcing function in the glucose dynamics.

### Glucose Rate of Appearance

As regards modeling the glucose rate of appearance, the description of the gastrointestinal tract proposed in the present work by means of a sequence of three compartments represents a simplification of the continuous one-dimensional process described by Salinari et al. [13]. While this latter type of modeling sounds physiologically appealing, model fitting requires the observation of GLP-1 concentrations, which are in fact never collected in standard clinical conditions, making the model difficult to use in practice. In fact, the poor performance of this model with the present series of patients (see below) may stem essentially from the fact that in our series GLP-1 determinations were not available. Other approaches [10], [16] are not as convincing from a physiological point of view, when the parametric model of the glucose rate of appearance (Ra) is described by a piecewise linear model without mechanistic biologic interpretation. A similar representation is indeed found in Silber et al. [26] where a “flexible input model” is used, that is an empirical model in which the input rate is modeled as a series of zero-order inputs.

### Insulin Release

Another fundamental feature introduced by the present model is the Hill dynamics used to represent insulin secretion, where the parameter *γ* in the function represents the non-linear pancreatic progression in insulin production as glucose concentrations rise. In the context of normal pancreatic reserve, at low glycemic levels a minority of the beta-cell population is actually secreting at high rates. If at baseline glycemia the relatively quiescent beta-cell subpopulation is large with respect to the active subpopulation, as glycemia increases a progressive recruitment of secretory units occurs. Conversely, in advanced stages of pancreatic insufficiency, a proportionally large beta-cell subpopulation is already active at baseline glycemic levels, and further increases in glycemia lead to quick saturation of the limited remaining secretion reserve. This “population-of-independent controllers” beta-cell recruiting mechanism, originally postulated by Grodsky [28], has in fact been found capable to explain both fast and slow experimentally observed insulin oscillations [29]. The *γ* parameter represents the “acceleration towards maximal values” of insulin secretion rate as glycemia and incretin hormones increase, and it is highest when relatively small increases in glycemia already push insulin secretion to near-maximal capacity. This is in fact what happens with T2DM patients, for whom residual insulin secretion reserve is so severely compromised that small increases in glycemia are already sufficient to obtain near maximal secretion. IFG and IFG+IGT together represent an intermediate situation, and, in this case also, saturation of pancreatic response could be due to glucose concentrations already high at baseline. For the NGT plus IGT group, residual reserve is large (due to normal baseline plus possible insulin hyper-secretion in IGT) and the *γ* coefficient is correspondingly small.

Of great interest in describing insulin release is also the maximal rate of insulin secretion 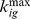. While ANOVA among patient groups was not statistically significant, the trend in the average values is consistent with our understanding of the physiology: the high-secretor groups are the two groups of subjects showing postprandial glucose intolerance, who hyper-secrete in response to the glucose load. The three groups with lower insulin secretion after oral glucose load are NGT and IFG (who have no problems disposing of the load) and T2DM (who may show relative secretion insufficiency, and for this reason show the frank clinical picture of diabetes).

It is commonly believed that some kind of pancreatic “exhaustion” underlies the eventual insulin secretion failure and the emergence of the frank picture of diabetes mellitus in the final stages of the pathogenesis of T2DM. The present OGTT model offers a numerical index *γ*, directly estimable from the standard clinical test, which quantifies the degree of pancreatic secretory insufficiency. A possible physiologic interpretation of variations of this index (based on the varying ability to recruit additional pancreatic secretory units) has been attempted above. Whether this interpretation is correct is open to debate, but the numerical values of this index, as estimated in progressively more severe stages in the development of the disease, are certainly suggestive that some essential mechanism has in fact been identified. The present modeling study, therefore, quantifies in this way the progression of disease, which is clearly associated with a progressive derangement of all indices of insulin sensitivity (whether empirical or model-derived), as well as with a progressive increase in average BMI.

Conversely, while empirical insulin secretion indices (HomaBCF, IGenic, AUCrig) show no statistical significance in differentiating groups with respect to secretory response, the progressive failure of insulin secretion is associated with a very significantly different SIMO *γ* model parameter, pointing to variations in pancreatic secretory reserve among the studied groups. The trend in the SIMO 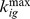
parameter, albeit not statistically significant, corroborates this interpretation.

### Pathophysiology and Insulin Sensitivity Indices

Panels A and B of Figures 2 show the differences in the dynamics of the glucose insulin system after orally administered perturbation in the five patient groups. First of all, there is a modest difference in postprandial glucose dynamics between the NGT and IFG groups, as can be expected, and actually some differences in the insulin secretion profile, due above all to a larger sample variability in the IFG group. Conversely, the glycemia profile worsens from NGT to IGT to IFG+IGT and then to T2DM, with a progressive slowing of the return to baseline levels and a progressive increase of the peak glycemic levels attained. The contribution of pancreatic secretory insufficiency to the pathogenesis of T2DM is well highlighted by the actual decrease of insulin secretion in the progression from IFG+IGT to T2DM.

All of the above pathophysiologic considerations are certainly not new, and agree with the trends observed for basal glycemia and basal insulinemia, which correspond with what is expected (with basal insulinemia decreasing in T2DM with respect to IFG+IGT, possibly due to progressive secretory defects), as well as with the trend of G_2h_ values, for which post-hoc comparisons highlighted the presence of three groups (NGT and IFG together, which were not significantly different from each other, but which were different from IGT and IFG+IGT subjects together, who in turn differed from T2DM subjects).

It is however of interest to see that the expected pathophysiology is faithfully mirrored in the proposed model’s parameters, as they change from group to group. In particular, the values of G_2h_ were strongly and inversely correlated with the SIMO insulin sensitivity index *k_xgi_*: high values of G_2h_ reflect, in fact, a defect in peripheral insulin action, translating into an impaired muscle glucose absorption mechanism.

In this context, explicit insulin secretion modeling serves several useful purposes: it provides a clear physiological description of the underlying mechanisms; it resolves statistical inconsistencies, by exhibiting a theoretical expected insulin time course around which observations scatter with error (for a thorough discussion of this problem see [17]); it determines a sensible improvement in data fitting in comparison with the piecewise-linear Ra models, in terms of the precision of parameter estimates (above all with respect to insulin sensitivity), without sacrificing goodness of fit. This last improvement in parameter estimation precision allows *k_xgi_* to decrease significantly from NGT, then to IFG, then to IGT, IFG+IGT and T2DM together, as is highlighted by post-hoc comparisons (Table 5). Conversely, when ANOVA was used to test differences among groups for the other two model-derived insulin sensitivity indices, no significance was obtained. Lack of significant differences for IS_COMO_ and IS_DMMO_ is in fact attributable to their high variability: both very large values (of the order of 10^−1^ for IS_COMO_ and of 10^−2^ for IS_DMMO_), and very small values (of the order of 10^−10^, the inferior limit of optimization, for IS_COMO_ and 10^−6^ for IS_DMMO_) were obtained from data fitting. It is to be noticed that a lack in the ability to differentiate groups is present also with IS_BREDA_, in spite of its overall strong correlation with the other insulin sensitivity indices.

Despite insulin sensitivity estimates way out of the acceptable range, however, both COMO and DMMO models seemed to perform well in terms of adapting predicted levels to observed plasma glucose concentrations. This is in effect a classical finding for over-parameterized models, where different parameter combinations may yield essentially indistinguishable data fits, making parameter identification imprecise. The bad performance of the COMO model could moreover be attributed to the lack of data related to GLP-1 measurements, making the problem of over-parameterization even worse.

Figure 3 summarizes this point very well: despite an apparently very good data fit, IS_COMO_ was estimated at 10^−10^ for an IFG subject and IS_DMMO_ was estimated at 3.26×10^−3^ (a very large value) for a T2DM subject; for these two subjects, SIMO (Figure 5 panels A2–A3 and B2–B3) estimated insulin sensitivity at respectively 3.24×10^−5^ and 4.58×10^−5^, well within physiologically plausible limits. It is clear that, in order to overcome over-parameterization for COMO and DMMO, and hence to reduce parameter variability, more observations could be used in the fitting procedure: the standard OGTT performed in clinical practice, however, yields glycemia and insulinemia observations over only seven time points.

Even when a sub-sample, not considering outliers (the “trimmed” sample), was considered, again ANOVA on IS_COMO_ and IS_DMMO_ resulted non-significant. Conversely, the results obtained (Table 5) with SIMO in both the full sample and the trimmed sample situations were quite similar.

The strongest positive correlations between well-known empirical insulin sensitivity indices and the three model-derived insulin sensitivity indices (IS_DMMO_, IS_COMO_ and k_xgi_) were obtained with *k_xgi_*; for IS_COMO_ in fact no correlation resulted significant whereas IS_DMMO_ showed weaker correlations.

It is interesting to observe that while IS_BREDA_ was more strongly correlated with all other indices (possibly because its definition involves the basic building elements common to most indices, AUC_G_ and AUC_I_), it was actually unable to significantly discriminate clinical groups with evidently different insulin sensitivities.

Another interesting finding is that if a completely naïf index is considered, (such as IS_NAIF_, defined simply as the inverse of average glycemia during the OGTT), this also correlates well with all other indices of insulin sensitivity and in fact discriminates significantly ALL clinical groups from one another. On one hand, this result underscores the fact that when we use a clinical classification based upon OGTT glycemias, it is not at all surprising that a simple summary of these glycemias is able to discriminate among the groups. Average glycemia, or equivalently glycemia AUC, or combinations of glycemias at relevant time points, all carry essentially the same information. On the other hand, it is clear that insulin resistance involves something more than just the glycemic levels attained. An index able to separate NGT from IFG and from the other three groups, while still assessing insulin sensitivity as approximately the same in IGT, IFG+IGT and T2DM, irrespective of the actual glycemias attained, could in fact be more informative about the underlying pathophysiology.

It may be worthwhile to clarify the criteria whereby we may judge an index to be “good”, in the absence of a gold standard (no one of the following criteria, taken by itself, should be determinant).

1. The index should be ***sound***: it should show a general correlation with similar indices. Clearly, insofar as a new index is worse than existing ones in characterizing the phenomenon under study, the correlation of the new with the old ones will be less than one. In fact, the correlation will also be less than one if the new index is better than existing ones in characterizing the phenomenon under investigation. Implied in the notion of soundness is the idea that the index values should be consistent with known phenomena: it cannot be, for example, that an index of insulin sensitivity assumes high values for known insulin-resistant subjects.
2. The index should be ***meaningful***, i.e. it should have a clear physiological meaning: it should, as far as possible, represent a clear (possibly simplified) component or mechanisms in the overall function under investigation. In any case, it should be clearly associated with the conceptual understanding of the phenomenon it seeks to quantify.
3. The index should be ***expressive***, in the sense that it should be able to discriminate experimental subjects or experimental situations expected to differ with regards to the phenomenon under consideration.
4. The index should be ***precise***, i.e. the numerical values it takes under the same experimental conditions should be concentrated around the expected theoretical value for those conditions. For example, repeated testing of the same subject should give rise to index values close to each other.
5. The index should be ***robust***, it should not vary widely upon modest variations of the data it is computed from. This criterion is somehow connected with the previous one: if an index is robust to small variations of the data, its realizations will be concentrated around the expected value upon repeated testing, hence it will be precise. An index is also robust when its computable value is relatively insensitive to missing data. In other words, an index, which can be computed to approximately the same value, notwithstanding some missing observations in the relevant dataset, is robust. It is to be noted that the requirements of robustness and expressivity are somehow in competition: a maximally robust index could be, in fact, a constant, completely insensitive to data variations, and for this reason totally inexpressive.
6. Ideally, the index should be ***accessible***: it should be simple to compute or a computation system for it should be readily available, so that it is not cumbersome to use and may be employed by a wide class of users at low cost (including the cost of the data necessary for its computation).

Applying these concepts to the set of indices studied in the present-work, we can see that all considered indices are sound, in the sense that they all exhibits positive correlations with one another. One exception is IS_COMO,_ whose main shortcoming, however, does not reside in the lack of soundness but in the lack of precision, from which the lack of correlation derives: on one hand the large number of parameters to be estimated with respect to the small number of observations, and on the other hand the need to fix the dynamics of GLP-1, given the lack of observed plasma GLP-1 concentrations, are the cause of its poor performance in terms of insulin sensitivity assessment for the present data set. The problem of over-parameterization, hence of lack of precision, is also evident with the IS_DMMO_ index, which, while being sound, is for this reason not expressive, since it is not able to discriminate at all experimental subjects coming from different pathophysiologic conditions. Both IS_COMO_ and IS_DMMO_ are model-derived indices, and they are as physiologically meaningful as the models from which they derive. For these two indices, the problem of over-parameterization also determines a lack of robustness: small variations in data or, even worse, an incomplete set of available observations determine wide swings in the numerical values these indices take (from 1.E-10 to 1.E-1 for IS_COMO_ and from 1.E-6 to 4.E-2 for IS_DMMO_), hence neither IS_COMO_ nor IS_DMM_ are robust indices. While IS_DMMO_ maintains the property of being rather accessible, as it derives from a parameter estimation procedure on data from a standard OGTT, IS_COMO_ is less accessible, since it requires GLP-1 measurements, which are not collected in standard clinical practice.

All the examined empirical indices (HOMA-IS, ISIcomp, MCR_est_, OGIS and IS_BREDA_), besides being sound, are accessible, precise and robust: they are in fact constructed from aggregated quantities, AUC_G_ and AUC_I_, which are not sensitive to small changes in observed data. While MCR_est_ and OGIS also result to be rather expressive, in our series HOMA-IS, ISIcomp and IS_BREDA_ were able to discriminate only between NGT and all the other groups combined. Apart possibly from the OGIS, whose construction is rather convoluted and reflects a number of rough simplifying assumptions, all the other empirical indices appear to be meaningful, since they all essentially express the concept that insulin resistance is definable as how much (supra-basal) insulin is necessary with respect to how much (supra-basal) glucose is circulating.

It is worth commenting on the IS_NAIF_ index, as an exercise in index-building. This index seems sound, in fact it correlates positively with the other indices. However, its formulation does not include any information related to insulin: there is therefore a likely lack of meaningfulness, and the apparent expressivity (it discriminates all groups from one another) derives from the fact that worsening glucose homestasis, as defined by glycemic levels (baseline and at 2 hours), implies by definition higher plasma glucose concentrations and correspondently lower IS_NAIF_ values. This index is obviously completely insensitive to changes in insulin concentrations, given its independence from it, and therefore its robustness and precision are completely artifactual. In fact, if an index is not meaningful, it is nonsense to consider all the other desirable characteristics as gauged from the performance of the index on a given dataset.

Finally, the *k_xgi_* index apparently complies with all the listed properties: it is sound due to its strong correlation with the other indices; it is meaningful due to its derivation from a model which reflects the essential physiological aspects of glucose homeostasis through insulin secretion and action; it is precise and robust (sample CVs are small in the five considered groups); it is expressive, discriminating among those groups, which are indeed expected to be different; it is accessible, in the same way as are all indices computable from the estimated parameters of a simple mathematical model of glycemia and insulinemia observations.

### Hepatic Glucose Output

The parameter *λ_1g_* in the hepatic glucose output function represents the decay rate with which liver glucose secretion is modeled to adapt directly to circulating plasma glucose and indirectly to circulating plasma insulin (through its effect on glycemia). This represents of course a simplification of the mechanisms by which liver glucose production is suppressed and glycogen-synthesis is enhanced in the presence of high plasma glucose and insulin concentrations. The results obtained for *λ_1g_*, even if not significant, show an interesting trend: the smallest estimate was obtained for the IFG group and the largest one for the IGT group. Results are consistent with central insulin resistance in the IFG condition. For IGT, it must be noticed that *λ*_1g_ expresses the sensitivity of Hepatic Glucose Output (HGO) to increases in glycemia only: since in IGT insulin is hypersecreted in response to glycemia, HGO appears more than normally suppressed for the same glucose concentrations.

### Final Comments

It is worth noting that the SIMO model is just the latest addition to several existing mathematical models for the glucose-insulin system. No model can of course be thought of as being definitive, and the present formulation only attempts to introduce a few meaningful improvements with respect to preexisting models.

The SIMO model is relatively simple, with only 7 free parameters to be estimated: this makes data fitting robust and provides acceptable precision in the estimates, even from a single-patient data set. In order to keep the structure simple and still be able to adequately fit experimental observations, this model explicitly recognizes the nonlinearity in the insulin secretion dependence on glycemia, and incorporates the effect of incretins, which stimulate insulin secretion upon oral glucose administration. Incretin effect and nonlinear insulin secretion progression are in fact the two innovative features that allow the model to realistically capture experimental curves of glycemia and insulinemia, without introducing empirical mathematical constructs with many degrees of freedom (which, while improving data fit, compromise both the robustness of the model and its ability to quantify the underlying physiology). The fact that the SIMO model could indeed meaningfully capture the relevant physiology would be supported by the regular and physiologically consistent change of its key parameter values along the spectrum of progressive metabolic decompensation states considered here. For this reason, and in conclusion, the use of the SIMO model for the interpretation of a subject’s OGTT may be practically informative to the clinician for the assessment of the type of lesion and of the stage of disease progression.

## Supporting Information

Table S1 **Medians of the Coefficients of variation of the SIMO parameter estimates on the whole sample, by group.**
(DOCX)

Table S2 **Medians of the Coefficients of variation of the DMMO parameter estimates on the whole sample, by group.**
(DOCX)

Table S3 **Means by group of the Insulin Sensitivity indices from the SIMO model and the DMMO model along with the Medians and the Interquartile Ranges (IQR) of the Coefficients of variation of the corresponding parameter estimates.** The first column reports results from the SIMO model on the whole sample (78 subjects); the second column reports results from the SIMO model on the 14 best subjects; the third column reports results from the DMMO on the 14 computable subjects.
(DOCX)

Table S4 **Descriptive statistics of the free model parameters of the SIMO model by group.**
(DOCX)

Table S5 **Descriptive statistics of the free model parameters and of the insulin sensitivity index SI_DMMO_ of the DMMO model by group.**
(DOCX)

Table S6 **Descriptive statistics of the free model parameters of the COMO model by group.**
(DOCX)

Appendix S1 **Description of the mathematical model of the Intestinal transit of glucose and incretin kinetics after the Oral Glucose Tolerance Test by Salinari et al.**
(DOCX)

Appendix S2 **Description of the Oral Glucose Minimal Model by Dalla Man et al.**
(DOCX)
