# Supplementary material for: Routine OGTT: A Robust Model Including Incretin Effect for Precise Identification of Insulin Sensitivity and Secretion in a Single Individual
Source: PLoS One. 2013 Aug 29;8(8):e70875. doi: 10.1371/journal.pone.0070875 (PMC3756988; doi:10.1371/journal.pone.0070875)
Supplement: Table S1 — Medians of the Coefficients of variation of the SIMO parameter estimates on the whole sample, by group. (DOCX) [file pone.0070875.s001.docx]

**Table S1. Medians of the Coefficients of variation of the SIMO parameter estimates on the whole sample, by group**

|  | **N** | **k_xgi_** | **f** | **k_xi_** | **γ** | **k_js_** | **k_gl_** | **λ** |
| --- | --- | --- | --- | --- | --- | --- | --- | --- |
| **NGT** | 28 | 9.24 | 169.61 | 9.06 | 18.30 | 43.13 | 52.80 | 503.86 |
| **IFG** | 15 | 10.56 | 159.49 | 9.48 | 18.32 | 29.70 | 134.24 | 386.96 |
| **IGT** | 13 | 11.92 | 294.75 | 7.80 | 32.87 | 49.96 | 58.02 | 62407.70 |
| **IFG+IGT** | 10 | 13.62 | 423.34 | 11.16 | 51.71 | 37.46 | 93.94 | 751.86 |
| **T2DM** | 12 | 16.93 | 3878.72 | 4.81 | 50.88 | 44.41 | 246.18 | 202.68 |
| **Whole sample** | 78 | 11.60 | 235.598 | 9.00 | 23.17 | 42.36 | 86.75 | 582.65 |
