# Supplementary material for: Routine OGTT: A Robust Model Including Incretin Effect for Precise Identification of Insulin Sensitivity and Secretion in a Single Individual
Source: PLoS One. 2013 Aug 29;8(8):e70875. doi: 10.1371/journal.pone.0070875 (PMC3756988; doi:10.1371/journal.pone.0070875)
Supplement: Table S2 — Medians of the Coefficients of variation of the DMMO parameter estimates on the whole sample, by group. (DOCX) [file pone.0070875.s002.docx]

**Table S2. Medians of the Coefficients of variation of the DMMO parameter estimates on the whole sample, by group**

|  | **N** | **SI** | **p_2_** | **p_3_** | **k_2_** | **k_3_** | **k_4_** | **k_5_** | **k_6_** | **k_7_** |
| --- | --- | --- | --- | --- | --- | --- | --- | --- | --- | --- |
| **NGT** | 6 | 15.79 | 43.19 | 30.20 | 119.35 | 413.26 | 566.25 | 793.43 | 235.07 | 319.65 |
| **IFG** | 2 | 9.05 | 48.65 | 12.63 | 23.03 | 189.33 | 735.31 | 48.94 | 935.88 | 5698.44 |
| **IGT** | 2 | 13.79 | 1185.04 | 232.12 | 115.26 | 1406.13 | 731.87 | 469.87 | 960.05 | 691.74 |
| **IFG+IGT** | 3 | 28.26 | 149.98 | 23.41 | 35.07 | 192.71 | 115.53 | 913.73 | 200.03 | 558.63 |
| **T2DM** | 1 | 50.77 | 54.31 | 16.23 | 82.19 | 233.93 | 67.69 | 51.55 | 239.87 | 735.67 |
| **Whole sample** | 14 | 19.98 | 93.76 | 21.63 | 73.51 | 279.40 | 237.92 | 504.77 | 238.70 | 505.25 |
