# Supplementary material for: Routine OGTT: A Robust Model Including Incretin Effect for Precise Identification of Insulin Sensitivity and Secretion in a Single Individual
Source: PLoS One. 2013 Aug 29;8(8):e70875. doi: 10.1371/journal.pone.0070875 (PMC3756988; doi:10.1371/journal.pone.0070875)
Supplement: Table S3 — Means by group of the Insulin Sensitivity indices from the SIMO model and the DMMO model along with the Medians and the Interquartile Ranges (IQR) of the Coefficients of variation of the corresponding parameter estimates. The first column reports results from the SIMO model on the whole sample (78 subjects); the second column reports results from the SIMO model on the 14 best subjects; the third column reports results from the DMMO on the 14 computable subjects. (DOCX) [file pone.0070875.s003.docx]

**Table S3. Means by group of the Insulin Sensitivity indices from the SIMO model and the DMMO model along with the Medians and the Interquartile Ranges (IQR) of the Coefficients of variation of the corresponding parameter estimates.**

The first column reports results from the SIMO model on the whole sample (78 subjects); the second column reports results from the SIMO model on the 14 best subjects; the third column reports results from the DMMO on the 14 computable subjects.

|  |  | **SIMO model** |  |  | **SIMO model** |  |  |  |  |
| --- | --- | --- | --- | --- | --- | --- | --- | --- | --- |
|  |  | **Whole Sample** |  |  | **Best 14** |  |  | **DMMO model** |  |
|  |  | **k_xgi_** | **CV -Median** |  | **k_xgi_** | **CV-Median** |  | **SI_DMMO_** | **CV-Median** |
|  | **N** | **Mean** | **(IQR)** | **N** | **Mean** | **(IQR)** | **N** | **Mean** | **(IQR)** |
| **NGT** | 28 | 8.624E-05 | 9.24 | 7 | 7.09E-05 | 4.76 | 6 | 2.03E-03 | 15.79 |
|  |  |  | (6.27-12.78) |  |  | (4.57-5.40) |  |  | (6.36-41.87) |
| **IFG** | 15 | 5.298E-05 | 10.56 | 1 | 2.97E-05 | 3.27 | 2 | 2.26E-04 | 9.05 |
|  |  |  | (8.27-12.88) |  |  | (3.27-3.27) |  |  | (8.23-9.86) |
| **IGT** | 13 | 2.092E-05 | 11.92 | 3 | 1.72E-05 | 5.57 | 2 | 5.02E-05 | 13.79 |
|  |  |  | (7.23-21.28) |  |  | (5.25-5.89) |  |  | (9.30-18.28) |
| **IFG+IGT** | 10 | 2.382E-05 | 13.62 | 2 | 2.57E-05 | 5.42 | 3 | 6.70E-05 | 28.26 |
|  |  |  | (7.67-17.91) |  |  | (5.41-5.42) |  |  | (24.07-30.20) |
| **T2DM** | 12 | 2.383E-05 | 16.93 | 1 | 1.83E-05 | 2.99 | 1 | 3.50E-04 | 50.77 |
|  |  |  | (12.19-18.61) |  |  | (2.99-2.99) |  |  | (50.77-50.77) |
