# Supplementary material for: Routine OGTT: A Robust Model Including Incretin Effect for Precise Identification of Insulin Sensitivity and Secretion in a Single Individual
Source: PLoS One. 2013 Aug 29;8(8):e70875. doi: 10.1371/journal.pone.0070875 (PMC3756988; doi:10.1371/journal.pone.0070875)
Supplement: Table S4 — Descriptive statistics of the free model parameters of the SIMO model by group. (DOCX) [file pone.0070875.s004.docx]

**Table S4. Descriptive statistics of the free model parameters of the SIMO model by group**

|  |  | **k_xgi_** | **f** | **k_xi_** | **γ** | **k_js_** | **k_gl_** | **λ_1g_** | *k_ig_^max^* | **T_ig_** |
| --- | --- | --- | --- | --- | --- | --- | --- | --- | --- | --- |
| **NGT** | **Mean** | 8.62E-05 | 0.6178 | 0.0633 | 4.4159 | 0.2891 | 0.1377 | 0.5857 | 45.93 | 0.8535 |
| **N = 28** | **Std. Deviation** | 9.36E-05 | 0.2282 | 0.0581 | 0.9609 | 0.3884 | 0.2494 | 0.3947 | 41.67 | 1.1988 |
|  | **Std. Error of Mean** | 1.77E-05 | 0.0431 | 0.0110 | 0.1816 | 0.0734 | 0.0471 | 0.0746 | 7.87 | 0.2265 |
|  | **Minimum** | 3.32E-07 | 0.2792 | 0.0124 | 2.3938 | 0.0083 | 0.0004 | 0.0100 | 9.30 | 0.0151 |
|  | **Maximum** | 0.000375 | 1 | 0.2 | 6.7885 | 1 | 1 | 1 | 164.76 | 4.3838 |
| **IFG** | **Mean** | 5.3E-05 | 0.6456 | 0.0361 | 6.5812 | 0.3333 | 0.1305 | 0.3871 | 34.21 | 1.5535 |
| **N = 15** | **Std. Deviation** | 5.18E-05 | 0.2007 | 0.0264 | 3.6865 | 0.4195 | 0.2056 | 0.4163 | 31.72 | 2.6356 |
|  | **Std. Error of Mean** | 1.34E-05 | 0.0518 | 0.0068 | 0.9518 | 0.1083 | 0.0531 | 0.1075 | 8.19 | 0.6805 |
|  | **Minimum** | 1.11E-05 | 0.4378 | 0.0000 | 4.1254 | 0.0294 | 0.0014 | 0.0100 | 6.88 | 0.0179 |
|  | **Maximum** | 0.000223 | 1 | 0.1134 | 19.561 | 1 | 0.85661 | 1 | 133.20 | 7.6742 |
| **IGT** | **Mean** | 2.09E-05 | 0.6435 | 0.0827 | 4.1549 | 0.2526 | 0.0408 | 0.7971 | 71.84 | 1.3210 |
| **N = 13** | **Std. Deviation** | 1.95E-05 | 0.2391 | 0.0781 | 0.8099 | 0.3573 | 0.0316 | 0.2556 | 66.52 | 1.5376 |
|  | **Std. Error of Mean** | 5.42E-06 | 0.0663 | 0.0217 | 0.2246 | 0.0991 | 0.0088 | 0.0709 | 18.45 | 0.4264 |
|  | **Minimum** | 4.15E-08 | 0.3196 | 0.0135 | 3.1444 | 0.0212 | 0.0017 | 0.3066 | 8.95 | 0.0429 |
|  | **Maximum** | 7.33E-05 | 1 | 0.2 | 5.3627 | 1 | 0.0916 | 1 | 186.54 | 5.2001 |
| **IFG+IGT** | **Mean** | 2.38E-05 | 0.7196 | 0.0600 | 6.2064 | 0.2896 | 0.1375 | 0.6041 | 72.02 | 1.7650 |
| **N = 10** | **Std. Deviation** | 2.28E-05 | 0.2372 | 0.0755 | 4.7626 | 0.4012 | 0.3044 | 0.3336 | 112.09 | 2.4975 |
|  | **Std. Error of Mean** | 7.2E-06 | 0.0750 | 0.0239 | 1.5061 | 0.1269 | 0.0963 | 0.1055 | 35.45 | 0.7898 |
|  | **Minimum** | 2.11E-06 | 0.2872 | 0.0001 | 2.3773 | 0.0141 | 0.0045 | 0.0100 | 2.04 | 0.0198 |
|  | **Maximum** | 7.18E-05 | 1 | 0.2 | 18.83 | 1 | 1 | 1 | 380.05 | 7.2992 |
| **T2DM** | **Mean** | 2.38E-05 | 0.7958 | 0.0729 | 7.9838 | 0.2985 | 0.2729 | 0.5827 | 43.06 | 7.6730 |
| **N = 12** | **Std. Deviation** | 2.09E-05 | 0.2554 | 0.0691 | 5.5061 | 0.4063 | 0.3625 | 0.3020 | 30.93 | 20.8409 |
|  | **Std. Error of Mean** | 6.02E-06 | 0.0737 | 0.0199 | 1.5895 | 0.1173 | 0.1047 | 0.0872 | 8.93 | 6.0162 |
|  | **Minimum** | 1E-10 | 0.2847 | 0.0128 | 2.9362 | 0.0110 | 0.0026 | 0.0140 | 6.79 | 0.0237 |
|  | **Maximum** | 6.99E-05 | 1 | 0.2 | 20 | 1 | 1 | 1 | 96.81 | 73.6560 |
