# Supplementary material for: Routine OGTT: A Robust Model Including Incretin Effect for Precise Identification of Insulin Sensitivity and Secretion in a Single Individual
Source: PLoS One. 2013 Aug 29;8(8):e70875. doi: 10.1371/journal.pone.0070875 (PMC3756988; doi:10.1371/journal.pone.0070875)
Supplement: Table S5 — Descriptive statistics of the free model parameters and of the insulin sensitivity index SIDMMO of the DMMO model by group. (DOCX) [file pone.0070875.s005.docx]

**Table S5. Descriptive statistics of the free model parameters and of the insulin sensitivity index SI_DMMO_ of the DMMO model by group.**

|  |  | **p_2_** | **p_3_** | **SI_DMMO_** | **k_2_** | **k_3_** | **k_4_** | **k_5_** | **k_6_** | **k_7_** |
| --- | --- | --- | --- | --- | --- | --- | --- | --- | --- | --- |
| **NGT** | **Mean** | 0.0234 | 2.86E-06 | 2.27E-03 | 2.706 | 2.728 | 2.143 | 2.823 | 1.489 | 0.381 |
| **N = 28** | **Std. Deviation** | 0.0321 | 2.81E-06 | 4.49E-03 | 2.510 | 2.921 | 1.759 | 1.958 | 1.223 | 0.474 |
|  | **Std. Error of Mean** | 0.0083 | 7.25E-07 | 1.16E-03 | 0.648 | 0.754 | 0.454 | 0.506 | 0.316 | 0.122 |
|  | **Minimum** | 0.0001 | 5.39E-07 | 3.31E-05 | 8.62E-42 | 2.94E-75 | 3.47E-37 | 3.61E-55 | 5.23E-46 | 1.41E-45 |
|  | **Maximum** | 1E-04 | 9.13E-06 | 1.47E-02 | 7.029 | 8.891 | 5.396 | 6.172 | 3.641 | 1.376 |
| **IFG** | **Mean** | 0.0579 | 1.83E-06 | 3.62E-04 | 2.942 | 2.802 | 2.581 | 1.909 | 1.301 | 0.576 |
| **N = 15** | **Std. Deviation** | 0.0441 | 2.25E-06 | 9.40E-04 | 2.410 | 2.319 | 1.717 | 1.891 | 1.032 | 0.632 |
|  | **Std. Error of Mean** | 0.0139 | 7.1E-07 | 2.97E-04 | 0.762 | 0.733 | 0.543 | 0.598 | 0.326 | 0.200 |
|  | **Minimum** | 1E-04 | 1E-07 | 1.00E-06 | 1.60E-18 | 4.46E-38 | 1.77E-82 | 4.31E-26 | 3.29E-66 | 1.74E-22 |
|  | **Maximum** | 0.1000 | 7.39E-06 | 3.03E-03 | 7.255 | 7.903 | 5.368 | 5.224 | 2.735 | 1.724 |
| **IGT** | **Mean** | 0.0375 | 1.76E-06 | 8.96E-04 | 1.925 | 3.126 | 2.041 | 2.327 | 1.898 | 0.396 |
| **N = 13** | **Std. Deviation** | 0.0415 | 2.16E-06 | 1.99E-03 | 1.859 | 3.008 | 2.205 | 1.708 | 1.607 | 0.713 |
|  | **Std. Error of Mean** | 0.0115 | 6E-07 | 5.51E-04 | 0.516 | 0.834 | 0.611 | 0.474 | 0.446 | 0.198 |
|  | **Minimum** | 1E-04 | 1E-07 | 1.01E-06 | 5.04E-64 | 1.39E-46 | 1.49E-32 | 1.28E-47 | 3.07E-44 | 1.89E-73 |
|  | **Maximum** | 0.1000 | 5.39E-06 | 7.10E-03 | 4.666 | 8.655 | 6.976 | 4.978 | 4.582 | 2.437 |
| **IFG+IGT** | **Mean** | 0.0304 | 3.87E-06 | 3.91E-03 | 2.016 | 2.348 | 2.072 | 2.018 | 1.633 | 0.828 |
| **N = 10** | **Std. Deviation** | 0.0342 | 3.2E-06 | 9.68E-03 | 2.306 | 2.631 | 2.032 | 1.542 | 1.179 | 0.891 |
|  | **Std. Error of Mean** | 0.0065 | 6.04E-07 | 1.83E-03 | 0.436 | 0.497 | 0.384 | 0.291 | 0.223 | 0.168 |
|  | **Minimum** | 1E-04 | 1.95E-07 | 3.27E-05 | 2.52E-52 | 2.52E-59 | 3.23E-35 | 6.52E-81 | 2.72E-49 | 5.15E-37 |
|  | **Maximum** | 0.1000 | 1.41E-05 | 3.64E-02 | 7.469 | 8.197 | 7.872 | 5.131 | 3.658 | 2.724 |
| **T2DM** | **Mean** | 0.0189 | 6.93E-07 | 1.32E-03 | 3.013 | 2.710 | 1.809 | 2.991 | 1.387 | 0.442 |
| **N = 12** | **Std. Deviation** | 0.0315 | 8.39E-07 | 2.16E-03 | 2.320 | 2.063 | 1.603 | 2.316 | 1.076 | 0.768 |
|  | **Std. Error of Mean** | 0.0091 | 2.42E-07 | 6.23E-04 | 0.670 | 0.596 | 0.463 | 0.669 | 0.311 | 0.222 |
|  | **Minimum** | 1E-04 | 1E-07 | 2.79E-06 | 1.04E-34 | 2.86E-68 | 9.39E-45 | 3.02E-50 | 6.17E-31 | 3.73E-99 |
|  | **Maximum** | 0.1000 | 2.54E-06 | 7.32E-03 | 7.680 | 5.749 | 4.693 | 6.751 | 3.541 | 2.508 |
