# Supplementary material for: Routine OGTT: A Robust Model Including Incretin Effect for Precise Identification of Insulin Sensitivity and Secretion in a Single Individual
Source: PLoS One. 2013 Aug 29;8(8):e70875. doi: 10.1371/journal.pone.0070875 (PMC3756988; doi:10.1371/journal.pone.0070875)
Supplement: Table S6 — Descriptive statistics of the free model parameters of the COMO model by group. (DOCX) [file pone.0070875.s006.docx]

**Table S6. Descriptive statistics of the free model parameters of the COMO model by group.**

|  |  | **c_2_** | **k** | **SG** | **SI** | **p** |
| --- | --- | --- | --- | --- | --- | --- |
| **NGT** | **Mean** | 31.04 | 0.0963 | 0.0128 | 3.31E-04 | 0.0367 |
|  | **Std. Deviation** | 43.63 | 0.2811 | 0.0180 | 1.14E-03 | 0.0603 |
|  | **Std. Error of Mean** | 8.25 | 0.0531 | 0.0034 | 2.15E-04 | 0.0114 |
|  | **Minimum** | 1.18 | 0.0012 | 1E-10 | 1E-10 | 3.54E-10 |
|  | **Maximum** | 140.45 | 1.51 | 0.0662 | 6.11E-03 | 0.2712 |
| **IFG** | **Mean** | 29.94 | 1.38 | 0.0093 | 1.06E-02 | 0.0246 |
|  | **Std. Deviation** | 38.01 | 3.22 | 0.0128 | 2.66E-02 | 0.0308 |
|  | **Std. Error of Mean** | 9.81 | 0.8307 | 0.0033 | 6.87E-03 | 0.0080 |
|  | **Minimum** | 0.38222 | 0.0074 | 1E-10 | 2.72E-05 | 5.38E-06 |
|  | **Maximum** | 119.80 | 11.75 | 0.0381 | 1.00E-01 | 0.0926 |
| **IGT** | **Mean** | 25.15 | 0.0412 | 0.0082 | 6.07E-03 | 0.0165 |
|  | **Std. Deviation** | 38.06 | 0.0214 | 0.0119 | 1.85E-02 | 0.0250 |
|  | **Std. Error of Mean** | 10.56 | 0.0059 | 0.0033 | 5.14E-03 | 0.0069 |
|  | **Minimum** | 2.67 | 0.0183 | 1E-10 | 1.01E-10 | 2.09E-10 |
|  | **Maximum** | 137.46 | 0.0892 | 0.0327 | 6.73E-02 | 0.0712 |
| **IGT+IFG** | **Mean** | 11.03 | 0.0461 | 0.0077 | 2.32E-04 | 0.1215 |
|  | **Std. Deviation** | 8.73 | 0.0232 | 0.0120 | 5.47E-04 | 0.3104 |
|  | **Std. Error of Mean** | 2.76 | 0.0073 | 0.0038 | 1.73E-04 | 0.0982 |
|  | **Minimum** | 1.13 | 0.0207 | 1.01E-10 | 1.01E-05 | 1.34E-10 |
|  | **Maximum** | 28.99 | 0.0909 | 0.0283 | 1.78E-03 | 1 |
| **T2DM** | **Mean** | 7.782792 | 0.0663 | 0.0273 | 2.71E-05 | 0.0092 |
|  | **Std. Deviation** | 3.53 | 0.0703 | 0.0111 | 5.83E-05 | 0.0179 |
|  | **Std. Error of Mean** | 1.02 | 0.0203 | 0.0032 | 1.68E-05 | 0.0052 |
|  | **Minimum** | 3.23 | 0.0255 | 0.0159 | 1.00E-10 | 1.19E-10 |
|  | **Maximum** | 16.14 | 0.2831 | 0.0513 | 1.84E-04 | 0.0625 |
