# Supplementary material for: Routine OGTT: A Robust Model Including Incretin Effect for Precise Identification of Insulin Sensitivity and Secretion in a Single Individual
Source: PLoS One. 2013 Aug 29;8(8):e70875. doi: 10.1371/journal.pone.0070875 (PMC3756988; doi:10.1371/journal.pone.0070875)
Supplement: Appendix S1 — Description of the mathematical model of the Intestinal transit of glucose and incretin kinetics after the Oral Glucose Tolerance Test by Salinari et al. (DOCX) [file pone.0070875.s007.docx]

**Appendix S1**

**The mathematical model of the Intestinal transit of glucose and incretin kinetics after the Oral Glucose Tolerance Test by Salinari et. al** [13]

For the analysis of OGTT data the formulation used by Salinari et. al is slightly different from that used by Dalla Man et al. when reporting the equations relating to the Glucose kinetics:

$\frac{dG\left(t\right)}{dt}=-[S_{G}+S_{I}Z\left(t\right)]G\left(t\right)+S_{G}Gb+\frac{Ra\left(t\right)}{V}$

$$G\left(0\right)=G_{b}$$

$$\frac{dZ\left(t\right)}{dt}=p\left(-Z\left(t\right)+[I\left(t\right)-I_{b}]\right)$$

$$Z\left(0\right)=0$$

where *S_G_* is glucose effectiveness, *S_I_* is insulin sensitivity, which is here a free parameter to be directly estimated, *p* is a rate constant, and *Z* is proportional to the variable *X* in the original formulation (*S_I_* = *p_3_/p_2_*, *p* = *p_2_*, *S_G_* = *p_1_*, *X* = *S_I_Z*).

The rate of appearance *Ra* is expressed by the following equation:

$$q\left(z,t\right)=\left\{\begin{matrix} q_{0}(t-z/u)\exp\left[-(1/u)\int_{0}^{z} \gamma\left(z'\right)dz'\right],\text{ }0\leq t-z/u\leq\theta,\text{ }z\geq0 \\ 0,\text{ otherwise} \end{matrix}\right.$$

with

$$q_{0}\left(0,t\right)=\left\{\begin{matrix} \left(1/u\right)\eta\left(t\right)\text{ }0\leq t\leq\theta,\text{ }z\geq0 \\ 0,\text{ }t\geq\theta\end{matrix}\right.$$

$\eta\left(t\right)=-D\left(\frac{dh\left(t\right)}{dt}\right)$ with $h\left(t\right)=\exp\left[\left(-kt\right)^{\beta}\right]$

where $\eta\left(t\right)$ [mmol/min] is the rate of glucose delivery to duodenum, *D* is the administered dose, $h\left(t\right)$ is the fraction of glucose retained in the stomach at time t after glucose ingestion.

$\gamma\left(z\right)$is the rate coefficient for the glucose absorption by the enterocytes along the gut lumen, depending on spatial position to account for a non-uniform regional distribution of glucose transporters. The Authors defined two space-dependent absorption rate coefficients, $\gamma_{1}\left(z\right)$and$\gamma_{2}\left(z\right)$, corresponding to the presence of two glucose transporters (one located in the upper small intestine, duodenum-jejunum, and the other in the lower small intestine, jejunum-ileum) proportional to Gaussian functions with means *z_1_* and *z_2_*, standard deviation *s_1_* and *s_2_* and proportionality coefficients *c_1_* and *c_2_*.

The Minimal Model equations for the analysis of OGTT data are completed with the equation of the GLP-1 kinetics:

**,** $GLP\left(0\right)=GLP_{b}$

where

where GLP is the plasma concentration of GLP-1, *GLP_b_* is the basal concentration, *a_GLP_* is the degradation rate constant, is the rate of glucose absorption, and where $\gamma_{L}\left(z\right)$, which is a rate coefficient, is assumed to have a proximal and a distal component to reflect the release of GLP-1 occurring both in the upper small intestine and at distal ileal sites. Moreover $\gamma_{L}\left(z\right)$ was set equal to $\gamma\left(z\right)$. The parameter *b_GLP_* represents the “efficiency” of the mechanism of GLP-1 secretion.
