# Supplementary material for: Routine OGTT: A Robust Model Including Incretin Effect for Precise Identification of Insulin Sensitivity and Secretion in a Single Individual
Source: PLoS One. 2013 Aug 29;8(8):e70875. doi: 10.1371/journal.pone.0070875 (PMC3756988; doi:10.1371/journal.pone.0070875)
Supplement: Appendix S2 — Description of the Oral Glucose Minimal Model by Dalla Man et al. (DOCX) [file pone.0070875.s008.docx]

**Appendix S2**

**The Oral Glucose Minimal Model by Dalla Man et al.** [10]

The model couples the classical minimal model of glucose kinetics [14] with a parametric description of the Rate of absorption *Ra*. The formulation of the oral minimal model is therefore:

$\frac{dG\left(t\right)}{dt}=[p_{1}+X\left(t\right)]G\left(t\right)+p_{1}Gb+\frac{Ra\left(t\right)}{V}$ $G\left(0\right)=G_{b}$

$\frac{dX\left(t\right)}{dt}=-p_{2}X\left(t\right)+p_{3}[I\left(t\right)-I_{b}]$ $X\left(0\right)=0$

where *G* is glucose concentration, *X* is insulin action, I is insulin plasma concentration, *Ra* is the glucose rate of appearance, *V* is the distribution volume, b denotes basal values, *p_1_*, *p_2_* and *p_3_* are rate parameters. The Insulin Sensitivity index SI is given by:

$$SI=\frac{p_{3}}{p_{2}}$$

and is expressed in min^-1^pM.

The Rate of Appearance *Ra* is described as a parametric function depending on a series of parameters. In the original work [10], the Authors proposed three different possible representations of *Ra*, suggesting that the Piecewise-Linear Model was more appropriate. Therefore, in the present work the Piecewise-Linear Model has been used. This representation describes *Ra* by means of a piecewise-linear function with a given number of break points at 0, 10, 30, 60, 90, 120, 180 minutes after glucose administration. The expression of *Ra* is therefore as follows:

$$Ra\left(t\right)=\left\{\begin{matrix} k_{i-1}+\frac{k_{i}-k_{i-1}}{t_{i}-t_{i-1}}\left(t-t_{i-1}\right),\text{ }t_{i-1}\leq t\leq t_{i},\text{ i=1...6} \\ 0\text{ otherwise} \end{matrix}\right.$$

where the set $\left\{k_{i}\right\}$is the unknown set of parameters to be estimated, representing the values of *Ra* at the break points. Parameter k_0_ was set to 0 since *k_0_*=*Ra*(0)=0. As suggested by the authors, the parameters *V* and *p_1_* were set at 0.17 *L/Kg* and at 0.014 min^-1^ respectively. Another constraint was finally introduced by the Authors because of a posteriori un-identifiability:

$$Ra\left(t\right)=\int_{0}^{180}Ra\left(t\right)dt=\frac{Df}{BW}$$

where *D* is the ingested dose, *f* is the fraction of the ingested dose that is actually absorbed, set at 0.86, and *BW* is body weight.
